# Classification of driver and passenger mutations in different cancer types using deep neural networks

**DOI:** 10.1093/bioadv/vbag068

**Published:** 2026-02-26

**Authors:** Medha Pandey, Anoosha Paruchuri, M Michael Gromiha

**Affiliations:** Department of Biotechnology, Bhupat and Jyoti Mehta School of Biosciences, Indian Institute of Technology Madras, Chennai, Tamil Nadu 600036, India; Comprehensive Cancer Center, James Cancer Hospital, The Ohio State University, Columbus, OH, 43210, United States; Department of Biotechnology, Bhupat and Jyoti Mehta School of Biosciences, Indian Institute of Technology Madras, Chennai, Tamil Nadu 600036, India

## Abstract

**Motivation:**

Cancer is driven by genetic changes, known as mutations, that lead to the uncontrolled division of cells. The functional significance of a vast number of these cancer somatic mutations is unknown, and it is one of the major challenges in cancer research. In this study, we performed an integrative analysis of 30 tumor types from PAN-cancer mutation data collected from the COSMIC database. We have analyzed a set of 61 364 missense mutations (57 535 drivers and 3829 passengers) from 682 cancer-causing genes and derived various important features from amino acid sequences, predicted AlphaFold structures, and amino acid contact networks. We observed that the motif-based preference, neighboring residue information, residue depth, and disorder regions around the site of mutation are important for the discrimination of drivers and passengers.

**Results:**

We further developed cancer-specific computational models to discriminate cancer-causing and passenger mutations using deep learning, and the integration of AlphaFold predicted structure information improved the pathogenicity prediction of mutations. Our method achieved an average classification accuracy of 84.06% with 10-fold cross-validation.

**Availability and implementation:**

The prediction server is available at https://web.iitm.ac.in/bioinfo2/PANDriver/index.html. We envisage that the AI-based prediction models would be an important tool to identify driver mutations and could extend the scope of precision medicine for cancer.

## 1. Introduction

Cancer is a leading cause of death worldwide, accounting for nearly 10 million deaths or one in six deaths. The most common cancers include breast, lung, colon, rectum, and prostate. Cancer statistics collected from the American Cancer Society suggest that the mortality rate due to cancer has decreased since 1991, but specific cancers, such as breast, prostate, and uterine cancer, have increased in incidence, which needs to be addressed (Siegel *et al.* 2023). Due to advancements in large-scale genomics and parallel sequencing technologies, a huge number of somatic mutations in various cancer types have become a powerful resource. These mutations with detailed clinical information are stored in major databases including The Cancer Genome Atlas (TCGA) (Weinstein *et al.* 2013), International Cancer Genome Consortium (ICGC) (Jennings and Thomas 2016), Database of Curated Mutations in cancer (DoCM) (Ainscough *et al.* 2016), and Catalogue Of Somatic Mutations In Cancer (COSMIC) (Tate *et al.* 2019). Various studies have utilized these large-scale mutation data for systematic analysis of cancer mutations, which is necessary to explore the preferred patterns of somatic mutations at the genome (Bozic *et al*. 2010, Abecasis *et al.* 2012, Iengar 2012, Kandoth *et al.* 2013, Vogelstein *et al.* 2013, Rentzsch *et al.* 2019, Oh and Sung 2021) and proteome levels (Mao *et al.* 2013, Anoosha *et al.* 2016, Pejaver *et al.* 2020, Pandey *et al.* 2022). While these analyses have provided insights into the complex landscape of mutations in cancer cells, the interpretation of individual mutations has not yet been completely explored. One of the key challenges is to identify functional mutations (drivers) from a large group of biologically neutral or non-functional mutations (passengers).

Several methods have been developed to identify functional/pathogenic mutations in various diseases (Thomas *et al.* 2006, Yue *et al.* 2019, Alabi *et al.* 2020, Tan *et al.* 2021, Lin *et al.* 2023) as well as specific to cancer (Tokheim and Karchin 2019, Chen *et al.* 2020, Pandey *et al.* 2022, Vyatkin *et al.* 2022). Anoosha *et al.* (2015) developed a supervised machine-learning model using support vector machines to discriminate cancer driver mutations from passengers, and the method is specific to the epidermal growth factor receptor (EGFR) protein, one of the most important targets in cancer. Pandey *et al.* (2022) proposed a machine-learning method to classify driver and passenger mutations associated with glioblastoma. Recently, deep learning has gained attention for its ability to handle large datasets and to provide more accurate predictions (Angermueller *et al.* 2016, Luo *et al.* 2019, Rogers *et al.* 2020, Liao *et al.* 2021, Ko *et al.* 2022).

Investigations have been carried out for individual proteins such as P53 and BRAF by integrating cancer-centric multi-omics data to identify specific patterns of mutations in different cancer subtypes (Mikhail *et al.* 2022, Selvam *et al.* 2023). Raimondi *et al.* (2021) described a procedure to define drivers and passengers across datasets to eliminate the dataset-construction bias in variant predictors and suggested considering all genes with driver and passenger variants. Wang *et al.* (2020) developed an ensemble method for identifying driver mutations based on 23 pathogenicity features. It predicts the driver status of mutations for somatic variants obtained from genome sequencing. Recently, Nourbakhsh *et al.* (2024) extensively reviewed available databases and prediction tools for driver genes and mutations.

Brandes *et al.* (2023) developed a generic genome-wide prediction for disease variant effects using a deep protein language model known as ESM1b. Zhou *et al.* (2023) developed another method for predicting the effects of mutations on protein stability using deep learning. Cheng *et al.* (2023) developed a classic deep-learning model, AlphaMissense, that predicts the pathogenicity of missense variants in proteins by assigning each variant a score indicating its likelihood of causing disease. While these methods have shown impressive performance in predicting variant effects, they are largely generic and not tailored to the specific mutational landscapes of individual cancer types. These models typically lack integration of cancer-specific context in terms of their mutational networks and the influence of neighboring residues, which are critical given the heterogeneity of cancer. In contrast, developing cancer-type-specific models allows for more precise identification of driver mutations by capturing the unique biological features of each cancer type, thereby improving relevance and interpretability.

In the current study, we have developed cancer-type-specific computational models using deep-learning-based neural network algorithms to classify driver and passenger mutations observed in 30 cancer types. Firstly, we grouped the driver and passenger mutations collected from different databases based on their frequency of occurrence and experimental annotations. We examined the preference of driver and passenger mutations in all cancer types and observed that E→K, R→Q, R→C and R→H substitutions are preferred in drivers, whereas A→T, P→L, P→S and V→I are preferred in neutral. We derived a set of 243 sequence, structure and network-based features and classified 61 364 mutants into drivers and passengers with an accuracy of 84.06% using 10-fold cross-validation. We observed that the neighboring residue information of the mutation site plays an important role in the discrimination of driver and passenger mutations. We suggest that these prediction results would serve as a significant tool in identifying the functional mutations in cancer and extend the scope of personalized treatment.

## 2. Methods

### 2.1 Construction of datasets

We collected mutation data for Cancer Gene Census from COSMIC (v97) (Tate *et al.* 2019), ClinVar (Landrum *et al.* 2018), Humsavar (https://www.uniprot.org/docs/humsavar), dbCPM (Yue *et al.* 2020), and dbSNP (Sherry *et al.* 2001) databases and segregated them into 30 distinct cancer types defined by TCGA. This allowed us to focus our analysis on genes that are closely associated with cancer and have been extensively studied in specific research endeavors. We screened this information from the pool of 13.6 million unique somatic missense mutations in COSMIC (v97), which are obtained from 320 886 patients. We obtained a total of 452 262 mutations for cancer driver mutations. To identify and eliminate redundancy, we employed Psi-CDHit (https://github.com/weizhongli/cdhit/blob/master/psi-cd-hit/README.psi-cd-hit) with a 40% identity cutoff and considered only one mutation if it occurs at the same position with the same substitution in different proteins of a cluster. For each cancer type, we checked for data availability and considered a mutation to be a driver if the sample count was ≥3 in COSMIC as a criterion to define driver mutations for each cancer type, which has been used in other studies in the literature (Ding *et al.* 2008, Jones *et al.* 2008, Anoosha *et al.* 2016, Tang *et al.* 2021). If a cancer type had ≥3000 mutations, we considered them individually. For example, breast carcinoma (BRCA) has 9130 mutations, and we developed a model specific to it. On the other hand, several cancer types have a very limited number of mutations (hundreds of data points). As these data are insufficient to train a reliable machine-learning model, we merged the mutations in these cancer types based on their origin so that they belong to a similar cancer type and have sufficient data.

On the other hand, we used the data available in the dbCPM and dbSNP databases to obtain neutral mutations. We compared the data in pathogenic and non-pathogenic mutations, and there is no overlap of mutations in these two classes. In the present study, the total number of driver mutations are 57 535, and the number of passengers is 3829. These numbers correspond to the unique number of mutations for all cancer types. We noticed that 11 258 driver mutations are present in different cancer types, and the total number of driver mutations is 68 793. For constructing the dataset for passenger mutations in each cancer type, first, we identified the genes that have driver mutations. Next, passenger mutations reported in these genes (among 3829) in the dbCPM and dbSNP databases are identified. These mutations are treated as passengers for each cancer type, and the number of passenger mutations is <3829 in any cancer type. Hence, passenger mutations in different cancer types are summed up to 39 264, whereas total unique passenger mutations are 3829.

We randomly split the datasets for each cancer type into 80:20 ratio, and among them, 80% of the data was used for training and 10-fold cross-validation. The remaining 20% data are used for test purposes as a blind dataset, which was never used in the training. Further, we segregated mutations from the same protein and kept them either in training or test, and evaluated the performance to avoid data leakage.

### 2.2 Computation of preferred driver and passenger mutations and odds ratio

The preference of driver and passenger mutations was calculated using the number of individual substitutions divided by the total number of drivers or passengers, i.e. *n*_ij_/*N* (where *n*_ij_ is the number of individual substitutions and *N* is the total number of drivers or passengers). We calculated the odds ratio to examine the preference of individual mutations toward drivers or passengers, which is calculated as the ratio of preferred driver and passenger mutations.


(1) $$\mathrm{Odds}\;\mathrm{ratio}=\left(n_{\mathrm{ijD}}/\;N_{D}\right)/\left(n_{\mathrm{ijP}}/\;N_{P}\right)\;,$$

where *n*_ijD_ and *n*_ijP_ are the number of individual substitutions in driver and passenger mutations, respectively, *N*_D_ and *N*_P_ are the total number of driver and passenger mutations, respectively.

### 2.3 Computation of sequence, structure and network-based features

#### 2.3.1 Sequence-based properties

We calculated 243 sequence-based features for the complete dataset. The features include physicochemical properties of amino acids, mutation matrices, preference of di and tri-peptide motifs (Pandey and Gromiha 2021, Pandey *et al.* 2022, Pandey and Gromiha 2023), position-specific scoring matrices (PSSM) profiles, conservation scores using AACon server (https://www.compbio.dundee.ac.uk/aacon/) (Valdar 2002, Troshin *et al.* 2018), and potentials linked to different substitutions from the amino acid index properties (Kawashima and Kanehisa 2000).

##### 2.3.1.1 Physicochemical properties

We have considered 152 properties belonging to physical, chemical, energetic, and conformational parameters in this study (Gromiha 2005). The change in property between the wild-type and the mutant residue is computed as:


(2) ΔP(mutation)=P(mutant) – P(wild-type),

where *P*(wild-type) and *P*(mutation) are the property values of wild-type and mutant residues, respectively, and Δ*P*(mutation) is the change in property due to mutation (Anoosha *et al.* 2015).

##### 2.3.1.2 AAindex mutation matrices and indices

We collected 22 amino acid mutation matrices and contact potentials from the AAIndex2 database (Kawashima *et al.* 2008), and the value is substituted for each mutation. Pair-wise contact potential matrices are collected from the AAIndex3 database (Kawashima *et al.* 2008), and the difference of amino acid contact potential for a mutation is obtained by subtracting the contact potential value of N-/C-neighbor of mutation position to wild-type residue from N-/C-neighbor to the mutant residue. We also added the influence of neighboring residues by calculating the difference between the left and right residues of the mutation position.

##### 2.3.1.3 Neighboring residue information

We have compiled a dataset of protein sequences for proteins associated with each cancer type from the UniProt database. As part of our analysis, we have considered the preferences of neighboring residues (N and C termini) in the protein sequence at the mutation position. Based on the window-length selection criteria from the previous study, we have considered a window length of 13 residues, which corresponds to 6 residues toward both the N terminus and the C terminus of the mutation site (Pandey and Gromiha 2021). To characterize the neighboring residues, we have classified the 20 amino acid residues into six categories based on the physicochemical properties of their side chains such as aliphatic, sulfur-containing, aromatic, polar, positively charged, and negatively charged, and computed the number of residues belonging to each category within the neighboring regions of the mutation site. This approach allows us to capture the influence of the local environment at the mutation position and provides valuable information about the composition of amino acid residues in different physicochemical categories within the proximity of the mutation site. We obtained six features using information about neighboring residues.

##### 2.3.1.4 Motifs identification

We further calculated seven di- and tri-peptide motif-based features using the site of mutation and residues present in the neighborhood by introducing gap(s). This method was adopted in our earlier studies (Pandey and Gromiha 2021, Pandey *et al.* 2022, Pandey and Gromiha 2023). We then calculated the odds ratio for each motif in drivers and passengers and used it as a feature.

##### 2.3.1.5 Position-specific scoring matrices

We extracted PSSM scores for all the proteins using Psi-BLAST and the Reference Proteome (Ref90) database downloaded from UniProt. PSSM scores capture the evolutionary information of each amino acid residue in a protein sequence by comparing it to a large set of homologous sequences. We extracted three different features using PSSM scores by (i) obtaining the average score in a window length of 13 around the mutation site, (ii) the difference between the scores of mutant and wild-type residues, and (iii) the PSSM score for the site of mutation. These extracted features provide valuable insights into the local sequence environment, including residue conservation, structural importance, and functional significance.

##### 2.3.1.6 Conservation scores

The AACon server (https://www.compbio.dundee.ac.uk/aacon/) is a powerful tool for calculating the conservation feature of amino acid residues in proteins. Conservation analysis plays a crucial role in understanding the functional and structural importance of residues in protein sequences. It employs various algorithms and methods to assess the conservation of amino acids across multiple sequence alignments and considers factors such as amino acid type, physicochemical properties, and evolutionary information to determine the degree of conservation for each residue. By utilizing the AACon server, we extracted 18 different conservation scores.

##### 2.3.1.7 Importance of disorder in mutations

We predicted sequence-based residue-wise disorder for all the proteins associated with different cancer types using IUPred2A (Erdős and Dosztányi 2020). which is a tool used for predicting intrinsically disordered regions in protein sequences. It is based on the observation that certain regions of proteins lack stable secondary or tertiary structures as well as exist in a disordered or flexible state.

#### 2.3.2 Structure-based properties

Amino acid changes are well known to change the structural integrity of proteins due to loss/gain of different interactions. We extracted the structures for all the proteins associated with driver and passenger mutations from the AlphaFold database (https://ftp.ebi.ac.uk/pub/databases/alphafold, 29 November 2021, v2) (Jumper *et al.* 2021). Structures are associated with many important features such as residue-wise centrality measures, accessible surface area, secondary structures, residue-wise interactions, and solvent accessibility. We used FEATURE (v3.1.0) framework, which uses a protein’s microenvironment to calculate physicochemical features (Halperin *et al.* 2008, Schmidt *et al.* 2023). For secondary structures and solvent-accessible surface areas, we used the software DSSP (Kabsch and Sander 1983). Inter-residue interactions and contacts were also extracted using a modified version of the Protinter software (https://github.com/Ax-Sch/protinter). The predicted local distance difference test (pLDDT) was extracted using the biopandas package. If several structural models were available for one protein, the average value for each feature across the structural models was used.

#### 2.3.3 Network-based properties

We used structures obtained from AlphaFold for calculating network-based properties. We developed a weighted amino acid network for each protein using the Python package biographs and a network with an atom–atom distance cutoff of 7 Å (https://github.com/rodogi/biographs). These networks were then used to calculate centrality measures such as closeness, betweenness, and eigenvalue centrality. We calculated four centrality-based features.

In the present study, most of the features are continuous, which were normalized between 0 and 1, except the secondary structure information, as this feature is discrete and we use one-hot encoding to define the secondary structure to be helix, strand, or coil.

### 2.4 Feature selection

We employed the selectkBest method to choose the features for each model. This approach to feature selection has been employed in prior studies for predicting protein structures, solubility, molecular chaperones, and stability (Fernandez-Escamilla *et al.* 2004, Pan *et al.* 2022). The “selectkBest” method is a feature selection technique used to choose the most relevant features from a dataset. It is part of the sklearn.feature_selection module in the popular scikit-learn library in Python. The method is used to reduce the dimensionality of the data by selecting the K (a user-defined number) best features based on some scoring function. This method chooses a scoring function that quantifies the importance or relevance of each feature and evaluates the relationship between each feature and the target variable. Common scoring functions include chi-square (for categorical targets), f-regression (for regression tasks), and mutual information (for both classification and regression tasks). The scoring function is then applied to each feature independently, assessing its relationship with the target variable. Higher scores indicate that a feature is more important or relevant to the target variable. Based on the scores obtained, the features are ranked in descending order. The feature with the highest score is considered the most relevant according to the scoring function. It retains only the top K features with the highest scores. This procedure was repeated up to 50 times to determine the optimal feature set, at which point the model’s performance reached a saturation point.

### 2.5 Performance evaluation

The model performance is evaluated using 10-fold cross-validation, and the classification performance of the model has been assessed by following measures:


(3) Sensitivity=TP/(TP+FN)

(4) Specificity=TN/(TN+FP)

(5) Accuracy=(TP+TN)/(TP+TN+FP+FN).

(6) Balanced accuracy=(sensitivity+specificity)/2

(7) MCC=(TP*TN-FP*FN)/√((TP+FP)*(TP+FN)*(TN+FP)*(TN+FN)),

where TP, TN, FP, FN, and MCC refer to the number of true positives, true negatives, false positives, false negatives, and Mathews Correlation Coefficient, respectively. Driver mutations are considered as a positive class, and passenger as negative class.

### 2.6 Deep-learning sequential model architecture

For the present study, we used Python’s keras library for developing sequential models for the classification of drivers and passengers in different cancer types. This library uses TensorFlow or Theano backend. These models are currently being used in pattern and speech recognition, histopathological image processing, computer vision, protein structure and function prediction, and genome engineering applications (Kim *et al.* 2017, Wen *et al.* 2017, Dildar *et al.* 2021, Liu *et al.* 2022, Schmidt *et al.* 2023). Further, several deep-learning implementations have been performed for cancer image classification, estrogen receptor status prediction, dermatologist-level classification, etc. (Esteva *et al.* 2017, Alakwaa *et al.* 2018, Wang *et al.* 2019). For simple numeric datasets, a simple architecture with fewer hidden layers, whereas for large and complex datasets, more hidden layers are required to abstract important features (Uzair and Jamil 2020).

The applied model can be explained by considering more than one hidden layer (*y*) that connects the input (*x*) and the output layer (*z*) with a weight (*W*) matrix as shown in (7). We used the sigmoid function as transitioning function.


(8) y=sigmoid(Wx+b).

In the next step, the transpose of the weight matrix (*WT*) is used to form the output layer *z*.


(9) $$z=\mathrm{sigmoid}\left(\mathrm{WTy}+b^{\prime }\right).$$

### 2.7 Model hyperparameters

We optimized a sequential model with a set of [1, 5] hidden layers, using henorm, relu, and softmax activations in different layers and sigmoid in the output layer. The model was compiled with the optimizer as Stochastic Gradient Descent, and the loss was evaluated using binary cross-entropy. We used model checkpoints such as callbacks and early stopping for efficient model training and robustness. Other hyperparameters such as momentum, regularizer, and learning rate were tuned to obtain final models. Finally, the output obtained from the last hidden layer was provided to a softmax classifier, which assigns new labels to the samples. These final labels are used to determine the class of the instance.

## 3. Results and discussion

### 3.1 Preference of driver and passenger mutations

**Figure 1:**
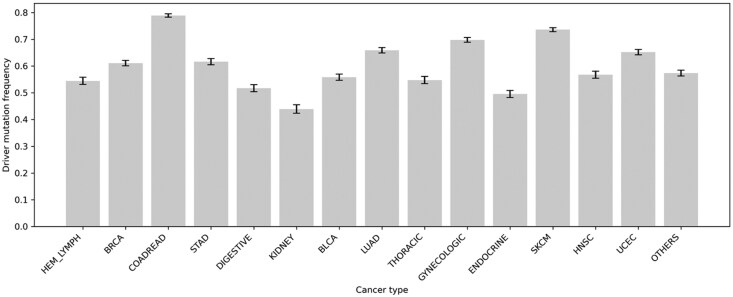
Driver mutation frequency across cancer types (Wilson 95% confidence interval).

The classification of all the cancer types into 15 subgroups showed that Colorectal carcinoma (COADREAD) had the highest number of driver mutations in 587 cancer-associated proteins (Fig. 1). On the other hand, kidney-related cancer subtypes (KICH, KIRC, and KIRP) and head and neck squamous carcinoma (HNSC) had comparatively smaller numbers of driver mutations (Supplementary Table S1). Other cancer types such as sarcoma (SARC), uveal melanoma (UVM), esophageal carcinoma, and testicular germ cell tumor have shown very less involvement of driver missense mutations. We also compared the distribution of the number of samples across cancer types from a pool of 320 886 patients and observed that Colorectal cancer (COADREAD) has the highest number of samples, followed by Endocrine, Lung (LUAD), Digestive, and Breast (BRCA) (Supplementary Fig. S1). This trend is similar to previously reported results on mutational landscape that BRCA, COADREAD, and LUAD are among the topmost dominant cancer types based on clinical data (Sinkala 2023). We also examined mutations annotated as “Pathogenic” in the ClinVar database (Landrum *et al.* 2018) across different disease types and found that breast cancer (BRCA) and colorectal cancer (COADREAD) rank among the top 10 disease categories. We have further analyzed the preference of proteins, which have a high number of driver mutations as well as associated with at least three cancer types. It showed a set of 629 proteins, and among them, 43 have more than 50 mutations, which include P53, PTPRD, CTNB1, BRCA1, and EGFR. The data are shown in Supplementary Table S2. We have also examined the list of proteins, where mutations occurred across the cancer types and observed that PTPRD, P53, NMDE1, MUC4, and BRCA1 are among the top 20 proteins with a higher number of mutations (Supplementary Fig. S2).

**Figure 2:**
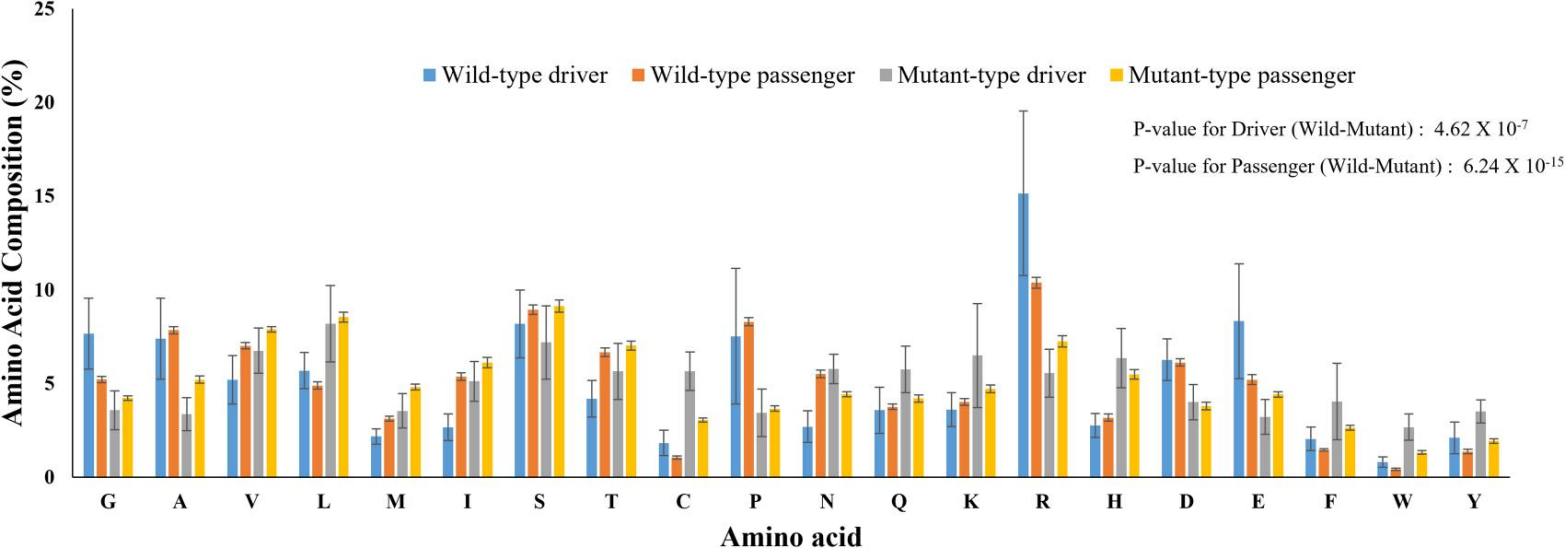
Distribution of amino acid residues in wild-type and mutant in driver and passenger mutations. Wild-type driver and wild-type passenger indicate the composition of amino acid residues in mutant sites (wild-type) of driver and passenger mutations, respectively. Similarly, mutant-type driver and mutant-type passenger are compositions of mutated residues (after mutation) in driver and passenger mutations, respectively. The data for each type is normalized to 100%. *P*-value was estimated using a Fisher’s exact test.

The distribution of amino acid residues in wild-type and mutant residues revealed that Arg, Glu, Ser, Gly, Ala, and Pro are frequently mutated in driver mutations (Fig. 2). Gly has unique structural properties that can influence the stability and function of proteins due to its flexibility and preference in turns or loops (David & Sternberg 2015). On the other hand, Arg and Glu are found in active sites or functional domains of proteins and participate in critical interactions or enzymatic activities (Petukh *et al.* 2015). The residues, Arg and Gly, are observed in many diseases as key contributors to disease (Vitkup *et al.* 2003). Mutations associated with Gly are associated with collagen-based diseases due to its presence in collagen structural motifs, genetic diseases, and in cancer types such as melanoma, renal cell carcinoma, and small cell lung carcinoma, etc. On the other hand, Arg is commonly observed to be substituted into His, Cys, Gln, and Trp due to its deamination of codons in 5’-CpG (Nelakurti *et al.* 2021).

We calculated the preference of driver and passenger mutations for a set of 61 364 missense mutations. Among the 380 substitutions, 224 were observed in drivers, and 159 were observed in passengers. We observed that E→K substitution is dominant in drivers followed by R→Q, R→C, R→H (Supplementary Table S3), whereas A→T, P→L, P→S, V→I, I→V were observed in passengers (Supplementary Table S4).

There are 72 unique driver mutations and 7 unique passenger mutations (K→A, A→L, W→V, R→A, Y→A, F→D, and W→P), whereas mutations such as A→T, P→L, P→S, and A→V are preferred in both driver and passenger mutations. On the other hand, some mutations are preferred either in driver or passenger mutations. For exploring such mutations, we calculated the odds ratio between driver and passenger mutants and observed that substitutions such as F→V substitution are dominant in drivers, followed by R→I, C→F, C→G, R→M, and R→T, whereas A→L, F→D, K→A, E→N, I→L, and H→L are preferred in passenger mutations (Supplementary Table S5).

We have examined the prevalence of the top 10 mutations in proteins with a higher number of driver mutations across different cancer types, and the results are summarized in Supplementary Table S6. Our analysis revealed that mutations such as E→K, A→T, R→Q, D→N, A→V, R→C, and E→D occur in at least three cancer types, consistent with the results previously reported in Anoosha *et al.* (2016).

### 3.2 Preference of motifs in driver and passenger mutations

We have analyzed the preferred motifs across cancer types and observed that each cancer type favors specific motifs, which shows the importance of these motifs and the necessity of cancer-specific methods to discriminate driver and passenger mutations. To examine the preference of motifs in driver and passenger mutations, we used a stringent cutoff of ≥1.2 and ≤0.8 for motifs that contribute to drivers and neutral mutations, respectively. For example, in hematopoietic lymphoid tissues (HEME LYMPH) motifs C**R**, E*I, G**F, S**R**L, and **Y**E are preferred in drivers, whereas S**M**, E*Y, D**K, R**R**S, **N**D, I*E, and T**N are preferred in passengers. Interestingly, motifs such as W**R**, R**C**, S**C**, T*E, R**F, T**P**P, **F**L, **H**H, and Q**Y are preferred in drivers, whereas W**M**, N**N, T**R**E, **N**Q, and I**M are preferred in passengers in at least three cancer types. The detailed list of preferred di- and tripeptides across different cancer types is presented in Supplementary Table S7.

### 3.3 Importance of feature selection

We have analyzed the importance of features for each cancer type, and the results are presented in Supplementary Table S8. Further, we have examined the features that are commonly selected in most of the cancer types. The analysis revealed that di and tri-peptide motifs, composition of negatively charged and polar residues, PSSM scoring values, conservation, physicochemical, energetic, and conformational properties, predicted local distance difference test AlphaFold structures, ASA, secondary structure, and residue depth occur commonly among different cancer types. However, the combination of these features is different for each cancer-type. On the other hand, centrality measures and residue-wise interactions are selected in limited cancer type subgroups including LUAD, Hem_Lymph, endocrine, and others. The top 20 features for three cancer-specific models SKCM, kidney, and HNSC are explained using SHAP analysis, and the results are shown in Supplementary Fig. S3. To address the impact of using AlphaFold’s predicted structure, we have compared the features obtained with X-ray and predicted structures. For a typical protein, p53, we obtained a correlation of 0.96, 0.92, and 0.93 for the properties, closeness centrality, secondary structure content, and surface area, respectively. The RMSD for the aligned structural region is 0.4 Å.

### 3.4 Discrimination of driver and passenger mutations

**Figure 3:**
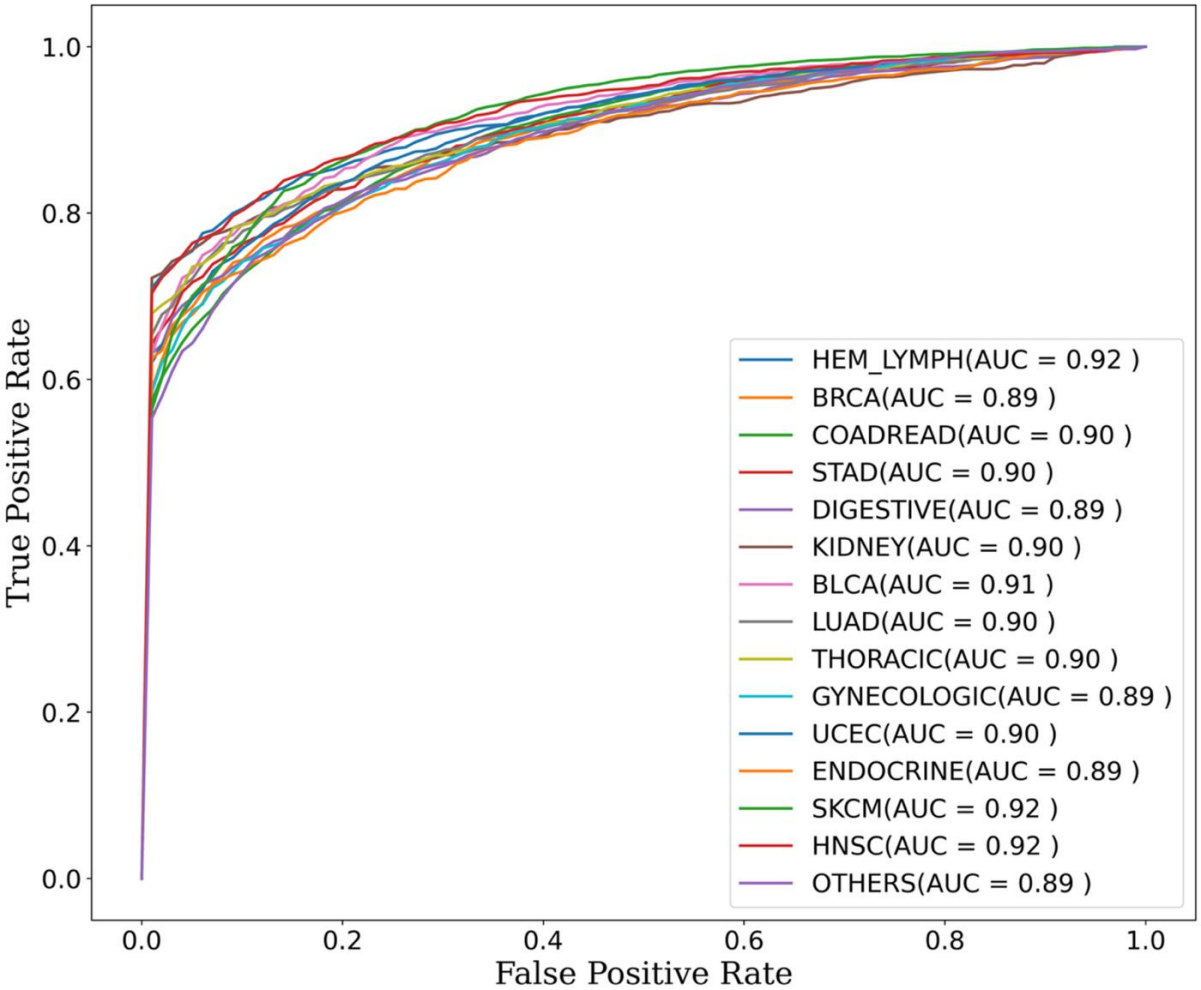
Area under the receiver operating characteristics curve (AU-ROC) for 10-fold cross-validation in different cancer types.

We have developed cancer-specific models using 80% of the initial data (Supplementary Table S1), and the results obtained with 10-fold cross-validation are presented in Table 1. We obtained an overall accuracy of 82.40% with 83.40% sensitivity and 75.75% specificity in 10-fold cross-validation. Interestingly, most of the models for different cancer types are able to achieve an area under the curve (AUC) of >0.8. The mean receiver operating characteristics curve (ROC) plot for 10-fold cross-validation of all cancer types is presented in Fig. 3.

**Table 1:** Model performance for training and 10-fold cross-validation.

Groups	Performance on training and 10-fold cross-validation					
	Sensitivity (%)	Specificity (%)	Accuracy (%)	Balanced accuracy (%)	AUC	MCC
Hem.Lymph	89.26 (82.39)	85.95 (84.76)	87.30 (83.49)	87.11 (83.57)	0.87 (0.92)	0.74 (0.68)
BRCA	83.51 (82.40)	83.23 (76.42)	83.40 (80.07)	83.37 (79.41)	0.83 (0.89)	0.67 (0.62)
COADREAD	81.33 (94.05)	84.15 (46.54)	81.93 (84.01)	82.74 (70.30)	0.83 (0.90)	0.57 (0.47)
STAD	83.78 (83.55)	86.66 (77.29)	84.89 (81.15)	85.22 (80.42)	0.85 (0.90)	0.69 (0.61)
Digestive	87.76 (77.13)	74.55 (85.40)	81.38 (81.13)	81.16 (81.27)	0.81 (0.89)	0.63 (0.63)
Kidney	73.97 (78.30)	98.99 (90.72)	88.00 (85.27)	86.48 (84.51)	0.86 (0.90)	0.77 (0.71)
BLCA	84.88 (79.71)	85.30 (85.51)	85.30 (85.51)	85.07 (82.27)	0.85 (0.91)	0.70 (0.65)
LUAD	88.29 (82.35)	80.57 (79.24)	85.66 (81.28)	84.43 (80.79)	0.84 (0.90)	0.61 (0.61)
Thoracic	77.03 (81.33)	95.29 (83.30)	85.29 (82.22)	86.16 (82.31)	0.86 (0.90)	0.72 (0.65)
Gynecologic	86.98 (84.49)	77.24 (70.94)	84.10 (80.39)	82.22 (77.71)	0.81 (0.89)	0.63 (0.55)
Endocrine	86.34 (88.00)	75.24 (66.34)	83.03 (81.45)	80.79 (77.17)	0.81 (0.89)	0.60 (0.56)
SKCM	94.42 (91.26)	68.01 (68.85)	87.45 (85.35)	81.22 (81.06)	0.81 (0.92)	0.66 (0.62)
HNSC	90.21 (83.71)	83.69 (85.60)	87.39 (84.53)	86.95 (84.66)	0.87 (0.92)	0.74 (0.61)
UCEC	95.19 (83.27)	58.61 (52.98)	82.46 (80.92)	76.90 (79.89)	0.77 (0.90)	0.60 (0.60)
Others	69.81 (79.03)	94.22 (79.63)	80.21 (79.28)	82.02 (79.33)	0.82 (0.89)	0.64 (0.52)
**Overall**	**84.85 (83.40)**	**84.88 (75.57)**	**84.52 (82.40)**	**83.45 (80.31)**	**0.83 (0.90)**	**0.66 (0.61)**

The values in parentheses represent the performance of 10-fold cross-validation. The number of features used for each cancer type varies from 25 to 37. Bold highlights the overall performance of the method.

We segregated mutations from the same protein and kept them either in training or test and evaluated the performance. Using the selected feature set for the models, we could achieve an average accuracy of 83.52% with a sensitivity and specificity of 82.18% and 84.48%, respectively. We have provided the performance of the method in the unbiased test set for each cancer type in Supplementary Table S9.

We further analyzed the driver mutations across cancer types and observed that 6843 mutations are common in at least three cancer types, and 48 817 mutations (77.1%) are unique. The predicted accuracy of common and unique mutations are 82.81% and 85.25%, respectively.

### 3.5 Performance of the method on a test set of mutants

**Figure 4:**
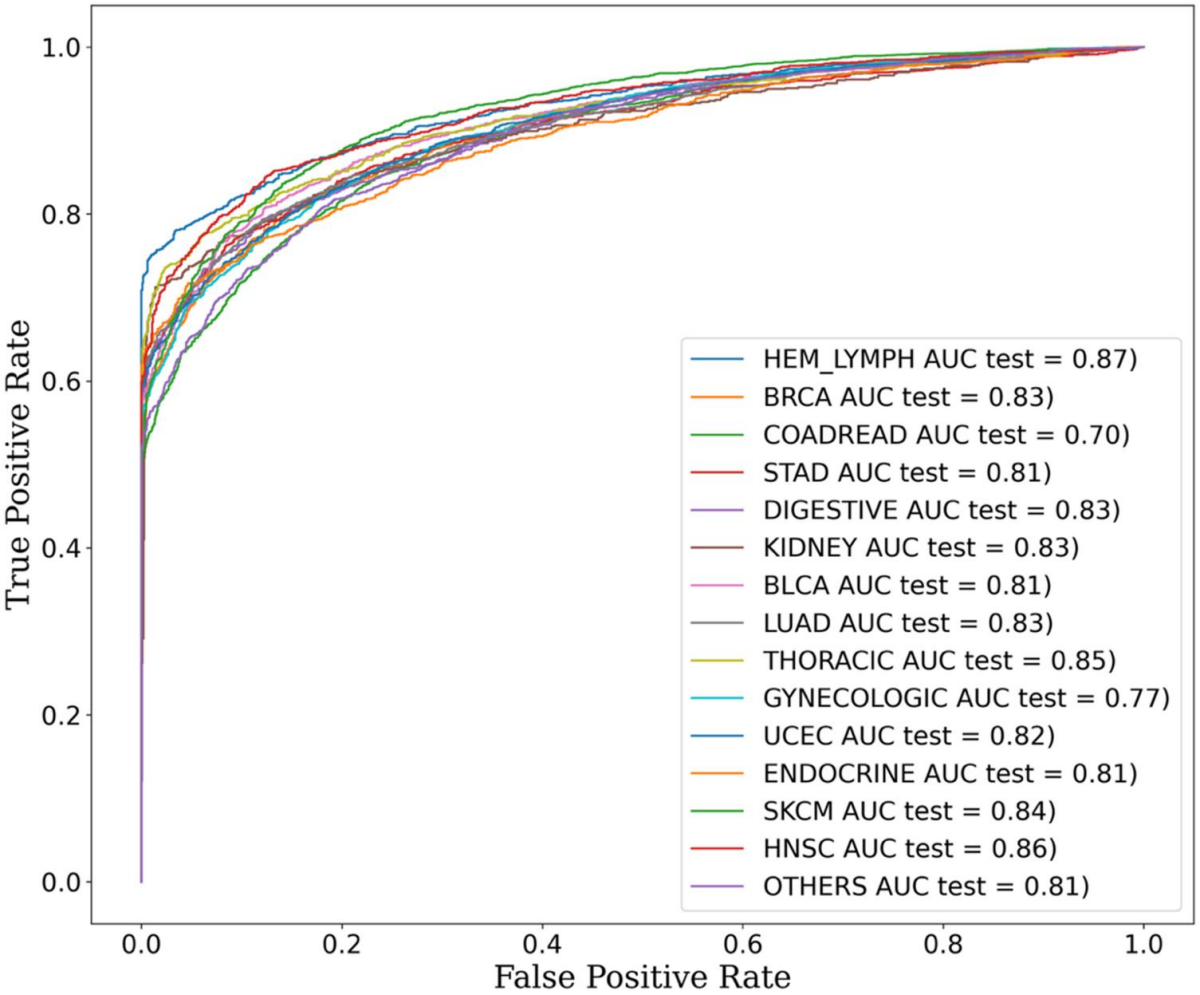
Area under the receiver operating characteristics curve (AU-ROC) for the test set across cancer types.

We have examined the performance of individual model with the respective test dataset of 20% mutants and observed that our method could classify driver and passenger mutations in each cancer type with an overall sensitivity, specificity, and accuracy of 83.94%, 78.02%, and 82.02%, respectively. The AU-ROC for most of the cancer types ranges between 0.70 and 0.87 and PR-AUC ranges between 0.92 and 0.97 (Table 2). ROC plots for the test results for all cancer types are presented in Fig. 4. The comparison between the AUC of the test and 10-fold cross-validation is shown in Supplementary Fig. S4.

**Table 2:** Model evaluation on test set for different cancer types.

Groups	Performance on training and 10-fold cross-validation						
	Sensitivity	Specificity	Accuracy	Balanced accuracy (%)	AUC	MCC	PR-AUC
	(%)	(%)	(%)				
Hem.Lymph	88.97	82.66	86.09	85.81	0.87	0.72	0.95
BRCA	81.1	82.26	81.55	81.68	0.83	0.62	0.94
COADREAD	80.74	81.68	80.94	81.21	0.7	0.54	0.97
STAD	80.5	82.93	81.43	81.71	0.81	0.62	0.94
Digestive	86.99	70.13	78.85	78.56	0.83	0.58	0.92
Kidney	72.42	98.07	86.8	85.24	0.83	0.74	0.93
BLCA	84.04	83.97	84.01	84	0.81	0.68	0.95
LUAD	84.9	77.35	82.33	81.13	0.83	0.61	0.96
Thoracic	74.09	92.88	82.59	83.49	0.85	0.67	0.94
Gynecologic	87.17	74.44	83.32	80.8	0.77	0.61	0.96
Endocrine	94.83	51.51	72.97	73.17	0.81	0.51	0.92
SKCM	93.66	63.45	85.68	78.55	0.84	0.61	0.97
HNSC	88.76	79.95	84.95	84.36	0.86	0.69	0.95
UCEC	93.57	55.72	80.4	74.65	0.82	0.55	0.96
Others	67.29	93.31	78.38	80.3	0.81	0.61	0.93
Overall	83.94	78.02	82.02	80.98	0.82	0.62	0.95

**Figure 5:**
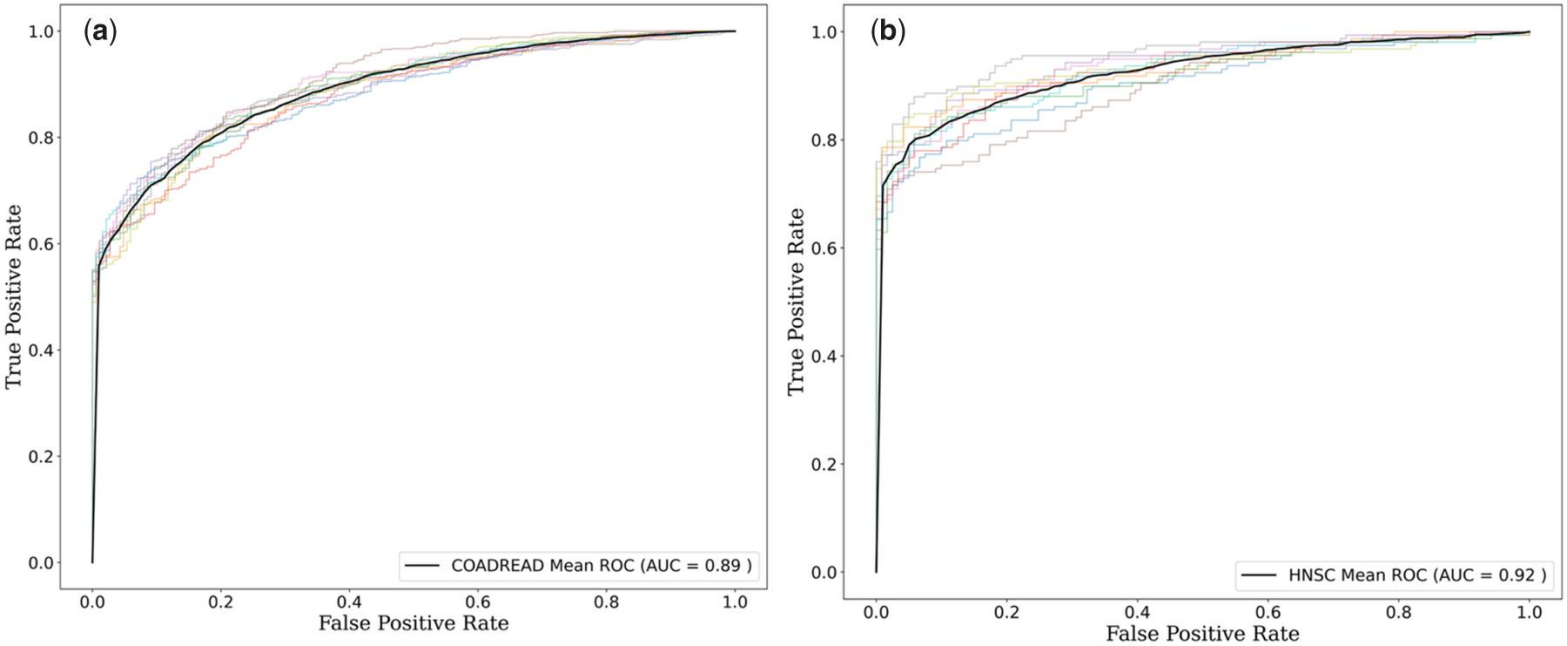
ROC plots for cancer types with (a) highest (COADREAD) and (b) lowest (HNSC) number of mutations obtained on 10-fold cross-validation.

We examined the performance of the present method for the cancer types with the highest and the lowest number of mutations. We found that COADREAD, with the highest number of drivers (13 953) and passenger (3735) mutations, showed an accuracy of 81.93% with an AUC of 0.89 in 10-fold cross-validation. On the other hand, HNSC had an accuracy of 87.39% with an AUC of 0.92 in 10-fold cross-validation. The ROC plots obtained on 10-fold cross-validation for these cancer types are presented in Fig. 5.

We noticed that most cancer types have the ratio of 1:1 to 1:2 between neutral and driver mutations [except Colorectal adenocarcinoma (COADREAD/READ*), gynecologic, and skin cancer (SKCM)]. Hence, we did not use any oversampling or undersampling to balance the data. On the other hand, our method efficiently handles such a dataset and shows a balanced sensitivity and specificity in most of the cancer types.

### 3.6 Performance of the model with frequently mutated genes

We have examined the prediction performance of our models on genes with the highest number of driver and passenger mutations, with a cutoff of 500 and 90 mutations, respectively, which include crucial genes such as P53, MUC4, DICER, and PTPRT (Table 3). P53 is one of the most common tumor suppressor genes commonly found in most of the cancer types, whereas MUC4 is commonly expressed in skin epithelium and acts as a ligand for receptor tyrosine kinase signaling (Carraway *et al.* 2009). These proteins can promote tumor growth by suppressing apoptosis and are observed in many cancer types including skin, lung, and ovarian cancer (Chen *et al.* 2022, Woldmar *et al.* 2023). Our models could predict driver mutations associated with these genes with a sensitivity ranging between 70% and 90%. We observed that the performance of discrimination improved for the genes such as TP53 with the second highest number of mutations, with 1215 drivers and 315 passengers, and the accuracy of discriminating them is 88.32%. For the other genes, the models correctly predicted the drivers and passengers at an accuracy ranging from 81%-86%.

**Table 3:** Proteins with the highest number of driver mutations.

UniProt ID	Gene name	Driver	Passenger	Sensitivity	Specificity	Accuracy
P23468	PTPRD	1276	–	85.99	–	–
P04637	P53	1215	315	90.66	85.39	88.32
Q99102	MUC4	793	135	81.50	82.96	81.66
P51532	SMCA4	681	105	83.77	73.33	82.73
Q9UPY3	DICER	568	90	83.85	77.78	83.19
P35916	VGFR3	561	210	86.47	80.48	85.11
Q8IXJ9	ASXL1	539	555	78.13	85.04	81.31

### 3.7 Performance of the model based on the number of mutations

We have analyzed the performance of the method based on the number of mutations in proteins. The rare mutations with a single driver mutation are present in genes NTM2B, SSX4, CREST, MYCL, ATF1, and RBTN2, which are observed in lung (LUAD), endocrine, skin (SKCM), colorectal (COADREAD), breast (BRCA), and others, respectively. Our method could correctly identify these driver mutations with a sensitivity of 86% (Table 4). We identified rare but potential drivers across different cancer types. Interestingly, our method is capable to rarely occurring driver mutations with a sensitivity of 85.71%. Further, for proteins with different numbers of mutations, it could classify the driver and passenger mutations with an accuracy ranging between 77% and 85.36% with an average sensitivity and specificity of 85.39% and 79.41%, respectively (Table 4).

**Table 4:** Performance of the models in proteins with different number of mutations.

No of mutations	No of proteins	Drivers	Passengers	Sensitivity	Specificity	Accuracy
1	21	21	–	85.71	–	85.71
2–10	142	776	318	86.34	73.90	82.72
11–20	78	1175	898	86.21	73.61	80.75
21–30	49	1257	1216	85.36	77.88	81.68
31–40	26	940	989	85.53	80.08	82.74
41–50	29	1383	1572	84.60	81.93	83.18
51–60	24	1441	1152	83.07	84.38	83.65
61–70	22	1550	1005	85.74	84.78	85.36
71–80	15	1283	1276	87.61	81.27	84.45
81–90	17	1569	1487	83.94	71.15	77.72
91–100	11	1140	908	85.35	81.61	83.69
>100	205	62935	32263	85.25	82.92	84.46

We have addressed the predictor “hotspot” bias in two directions: (i) examined the performance using the genes with less number of mutations. We noticed that 163 genes have less than 10 mutations, and our method can predict the driver and neural mutations with a sensitivity, specificity and accuracy of 86.02%, 73.90% and 84.21%, respectively and (ii) utilized the mutant sites with only one mutation and evaluated the performance. Our analysis showed that 44 635 sites are unique and the present method could distinguish driver and neutral mutations with an accuracy of 86.56, respectively.

We also examined the overlap of mutations across different cancer types in our dataset. It has 46,873 unique driver mutations, with no overlap between cancer types, and our method successfully predicted with an average sensitivity of 84.22%. Further, we analyzed a set of 10 662 driver mutations that are shared by at least two cancer types, which were predicted with an average sensitivity of 87.01%.

### 3.8 Performance of the model on experimentally validated mutation data

We evaluated the performance of the proposed model with experimentally validated mutation data containing 130 driver mutations collected from the literature (Kamburov *et al.* 2015). We segregated these mutations based on the associated cancer types. If any mutation is associated with more than one cancer types, we predicted using each associated cancer-specific model. We further compared the results for these drivers from the other state-of-art methods, which gave higher balanced accuracy with our test data and observed that the sensitivity in FATHMM-cancer, LIST-S2, and PROVEAN ranges between 27% and 75%. In contrast, our cancer-type-specific models can predict most of the drivers with an average sensitivity of 81.62%. The results for each cancer-type specific model are represented in Table 5. The model performed well on the known driver mutation data from different genes, and we suggest that these models could be used confidently in identifying a new driver mutation for a given sequence for specific cancer types. A few typical examples are discussed below.

**Table 5:** Performance of the model on experimentally predicted drivers and comparison with other state-of-art methods.

Cancer group	Number of proteins	Number of driver mutations	Number of predicted drivers	Comparison with other methods		
				FATHMM-cancer	LIST-S2	PROVEAN
BRCA	11	23	19 (82.60)	15 (65.21)	17 (73.91)	17 (73.91)
SKCM	5	15	13 (86.66)	9 (60)	8 (53.33)	7 (46.67)
LUAD	30	49	45 (91.83)	30 (61.22)	23 (49.94)	23 (49.94)
Digestive	3	11	6 (54.54)	8 (72.72)	3 (27.27)	5 (45.45)
Gynecologic	39	61	49 (80.32)	38 (62.29)	30 (49.18)	45 (73.77)
Thoracic	6	36	31 (86.11)	27 (75)	23 (63.89)	20 (55.55)
Others	52	88	79 (89.77)	61 (69.31)	61 (69.32)	59 (67.04)

R175H substitution in TP53, a tumor suppressor gene, is known to be involved in cancer by disrupting the interactions between the protein and DNA, interrupting the TP53 pathway that normally leads to apoptosis and resulting in accumulation of cells and tumor development (Pain *et al.* 2018). Another example, T790M mutation in EGFR, an oncogene, is a known functional mutation resulting in over-expression of the protein by altering the affinity of ATP binding to the protein kinase domain, leading to cell proliferation and tumor formation (Yu *et al.* 2018). This also leads to a change in the binding affinity of the protein to other ligands and affects the interactions of residues at the mutation site and their neighboring residues of the EGFR protein (Engel *et al.* 2016). These driver mutations are correctly identified by our method.

### 3.9 Performance of our method on oncogenic loss-of-function mutations

We evaluated our model on a curated set of 68 oncogenic loss-of-function (LoF) mutations (TP53: 59, PTPRT: 9) annotated in OncoKB (Chakravarty *et al.* 2017), which provides robust functional annotation across actionable cancer genes. Our model successfully predicted 51 of these mutations as drivers, corresponding to a sensitivity of 86.76%. This performance suggests that despite the recurrence-based selection strategy, our model can capture functionally important dispersed LoF events.

### 3.10 Comparison with existing methods

We obtained prediction results from 22 state of computational variant effect predictors from dbNSFP database (Liu *et al.* 2020) to assess the performance of our model on a common dataset of 9995 mutations (5639 drivers and 4320 passengers) from our test data (Table 6). The list includes SIFT (Kumar *et al.* 2009), PolyPhen-2 (Adzhubei *et al.* 2010), and DANN (Quang *et al.* 2015), which predict the non-synonymous amino acid mutations based on structure and functions as well as cancer-specific methods such as VEST4, Meta (SVM, LR, and RNN), and List-S2. We observed that MutationAssessor, CADD, FATHMM-MKL, and MVP showed very high sensitivity ranging between 86%-96%, whereas these methods have very less specificity. The cancer-specific method VEST4 showed an accuracy of 60.79% with sensitivity and specificity of 73.08% and 47.72%, respectively. We have systematically compared the test dataset against the precomputed AlphaMissense scores (Cheng *et al.* 2023). We observed that AlphaMissense was able to classify major drivers correctly with a sensitivity of 86% and 59% specificity. Our present method is specifically trained based on cancer-specific mutations, which leads to improved sensitivity and specificity. Further analysis showed that these methods are predicted with large false positives, which lead to less specificity. Our model showed an overall and balanced accuracy of 83.02% and 82.85%, respectively, with 85.59% sensitivity and 80.11% specificity on a large test dataset containing 30 different cancer types. It might be due to the training of a large number of balanced data for cancer and neutral mutations.

**Table 6:** Comparison of the present method with existing methods on test dataset.

Name	Computational method	Sensitivity	Specificity	Accuracy	Balanced accuracy
SIFT	Probabilistic; sequence homology; alignment	62.54	58.11	60.32	60.33
MutationTaster	Naïve Bayes classifier	86.17	33.55	61.10	59.86
FATHMM	Hidden Markov Model	35.45	53.90	44.06	44.67
MutationAssessor	Conservation-based statistical model	82.66	33.52	59.27	58.09
PROVEAN	Alignment-based delta score	53.94	67.29	60.12	60.62
VEST4	Random forest	73.30	45.71	60.04	59.51
MetaSVM	Support vector machine	38.56	71.96	54.15	55.26
MetaLR	Logistic regression	40.90	67.40	53.22	54.15
MetaRNN	Recurrent neural network	60.37	60.62	60.36	60.49
M-CAP	Gradient boosting	76.55	29.29	54.13	52.92
DEOGEN2	Gradient boosting	46.46	64.10	54.56	55.28
LIST-S2	Logistic regression (Ensemble)	74.90	47.29	61.73	61.10
MutPred	Random forest	52.98	62.33	57.18	57.65
MVP	Deep neural network	78.96	32.60	56.33	55.78
MPC	Bayesian/statistical score	78.44	55.12	67.19	66.78
Polyphen2	Naïve Bayes classifier	68.38	52.19	60.80	60.28
REVEL	Random forest; Ensemble; score from multiple predictors	41.92	65.16	52.81	53.54
LRT	Likelihood ratio test	71.53	47.15	60.43	59.34
CADD	Support vector machine	96.98	9.90	54.85	53.44
FATHMM_MKL	Multiple kernel learning	89.62	21.42	57.56	55.52
FATHMM-XF	Extreme boosting (XGBoost)	72.95	54.46	64.78	63.71
DANN	Deep neural network; genomic annotations	96.72	10.36	54.91	53.54
AlphaMissense	Deep neural network; protein language model; AlphaFold based	86.11	59.23	70.00	72.67
Present method	Deep neural network; protein sequence, structure, and network based	**85.59**	**80.11**	**83.02**	**82.85**

The bold values in the table represent the sensitivity, specificity, accuracy and balanced accuracy of the present method.

### 3.11 Clinical interpretation of driver mutations

We explored the cancer-specific TCGA datasets available in cBioPortal for overall cancer survival such as BRCA, COADREAD, STAD, BLCA, LUAD, and SKCM. We selected all proteins that are included in our dataset for the present study. This whole dataset comprised 2501 patients, and 2512 samples, and the overall survival (OS) is reported in Supplementary Fig. S5a. We observed that disease recurrence precedes mortality with the mutations associated with these proteins.

We further examined proteins harboring a high number of predicted driver mutations and evaluated their associated OS, as shown in Supplementary Fig. S5b. Notably, proteins such as **TP53, MUC4, KRAS, and PIK3CA**, which exhibit a higher burden of predicted driver mutations, were associated with poorer OS. These observations suggest that proteins enriched for predicted driver mutations tend to be linked with increased patient mortality.

## 4. Conclusions

The heterogeneous nature of different cancer types plays a significant role in the impact of driver mutations. Most of the cancer types exhibit a more complex genomic landscape with a larger number of diverse driver mutations, where proteins such as TP53, EGFR, and KRAS are very common. In recent years, the development of deep neural network tools has provided a wide opportunity to abstract high-level features using multiple layers for the prediction of different classes. Identification of features specific to cancer type and development of cancer-specific models can be a powerful method to predict potential driver mutations. Also, along with the other physicochemical properties, neighboring residue information, and the advent of the AlphaFold assisted with structure-specific properties such as residue depth, ASA, and residue-specific centrality have been proven crucial in discriminating drivers and passengers.

In this work, we implemented a deep neural network algorithm and developed multiple classification models for each cancer type to identify driver and passenger mutations, which can be used over a wide array of mutations from different genes at a single run. Our model showed an accuracy of 84.06% with a 10-fold cross-validation method and 83.56% accuracy on blind test data, which performed better than existing methods in the literature. In the comparison of 22 existing variant effect predictors, including both general and cancer-specific methods, our cancer type-specific model achieved a balanced accuracy of 82.85%, highlighting the advantage of tailored training on balanced, cancer-specific datasets. Our cancer-type specific models demonstrated high sensitivity (81.62%) on experimentally validated driver mutations across multiple genes and cancer types, supporting their utility in reliably identifying novel driver mutations in a cancer-specific context. Our method effectively addresses hotspot bias by maintaining high accuracy and sensitivity across low-frequency genes, unique mutation sites, and both cancer-specific and shared driver mutations.

Clinical interpretation of driver mutations integrates genomic recurrence, functional context, and evidence from clinical cohorts to link specific alterations with diagnosis, prognosis, and therapy response. Numerous studies demonstrate that driver mutations individually or in aggregate are associated with disease aggressiveness, survival outcomes, and treatment sensitivity in a cancer-type-dependent manner. Thus, identifying and contextualizing driver mutations provides clinically actionable insight into tumor biology and patient outcomes.

Key drivers such as TP53, KRAS, PIK3CA, and BRCA1/2 are known to influence tumor behavior and patient outcomes, with TP53 frequently associated with aggressive disease and reduced survival, and pathway-specific oncogenic drivers informing molecular subtypes and treatment strategies. In our analysis, genes harboring a higher burden of predicted driver mutations were associated with poorer OS, supporting their clinical relevance as markers of adverse prognosis. Together, these findings suggest that integrating driver mutation identification with clinical outcome data can aid in stratifying disease risk and interpreting mutation combinations in a clinically meaningful context.

Understanding the heterogeneity of cancer types and the impact of driver mutations is essential for personalized medicine approaches, as it enables the identification of specific molecular targets and the development of targeted therapies that exploit the vulnerabilities driven by these mutations. Moreover, comprehensive genomic profiling and large-scale cancer genomics initiatives have provided valuable insights into the heterogeneity and complexity of different cancer types, facilitating the identification of novel driver mutations and potential therapeutic strategies.

## Supplementary Material

vbag068_Supplementary_Data

## Data Availability

The dataset for each cancer type and models that support the findings of this study are openly available at https://github.com/medhapandey63/PANDriver.git. The repository includes all scripts used for feature extraction, details of the tools and Python packages employed, and instructions for running the cancer-specific models.

## References

[vbag068-B1] Abecasis GR , Auton A, Brooks LD et al An integrated map of genetic variation from 1092 human genomes. Nature 2012;491:56–65. 23128226 10.1038/nature11632 PMC3498066

[vbag068-B2] Adzhubei IA , Schmidt S, Peshkin L et al A method and server for predicting damaging missense mutations. Nat Methods 2010;7:248–9. 20354512 10.1038/nmeth0410-248 PMC2855889

[vbag068-B3] Ainscough BJ , Griffith M, Coffman AC et al DoCM: a database of curated mutations in cancer. Nat Methods 2016;13:806–7. 27684579 10.1038/nmeth.4000 PMC5317181

[vbag068-B4] Alabi RO , Elmusrati M, Sawazaki-Calone I et al Comparison of supervised machine learning classification techniques in prediction of locoregional recurrences in early oral tongue cancer. Int J Med Inform 2020;136:104068. 31923822 10.1016/j.ijmedinf.2019.104068

[vbag068-B5] Alakwaa FM , Chaudhary K, Garmire LX. Deep learning accurately predicts estrogen receptor status in breast cancer metabolomics data. J Proteome Res 2018;17:337–47. 29110491 10.1021/acs.jproteome.7b00595 PMC5759031

[vbag068-B6] Angermueller C , Pärnamaa T, Parts L et al Deep learning for computational biology. Mol Syst Biol 2016;12:878. 27474269 10.15252/msb.20156651 PMC4965871

[vbag068-B7] Anoosha P , Huang LT, Sakthivel R et al Discrimination of driver and passenger mutations in epidermal growth factor receptor in cancer. Mutat Res 2015;780:24–34. 26264175 10.1016/j.mrfmmm.2015.07.005

[vbag068-B8] Anoosha P , Sakthivel R, Gromiha MM. Exploring preferred amino acid mutations in cancer genes: applications to identify potential drug targets. Biochim Biophys Acta 2016;1862:155–65. 26581171 10.1016/j.bbadis.2015.11.006

[vbag068-B9] Bozic I , Antal T, Ohtsuki H et al Accumulation of driver and passenger mutations during tumor progression. Proc Natl Acad Sci U S A 2010;107:18545–50. 20876136 10.1073/pnas.1010978107 PMC2972991

[vbag068-B10] Brandes N , Goldman G, Wang CH et al Genome-wide prediction of disease variant effects with a deep protein language model. Nat Genet 2023;55:1512–22. 37563329 10.1038/s41588-023-01465-0 PMC10484790

[vbag068-B11] Carraway KL , Theodoropoulos G, Kozloski GA et al Muc4/MUC4 functions and regulation in cancer. Future Oncol 2009;5:1631–40. 20001800 10.2217/fon.09.125 PMC2825673

[vbag068-B12] Chakravarty D , Gao J, Phillips SM et al OncoKB: a precision oncology knowledge base. JCO Precis Oncol 2017;2017 PO.17.00011. 10.1200/PO.17.00011 PMC5586540 28890946

[vbag068-B13] Chen H , Li J, Wang Y et al Comprehensive assessment of computational algorithms in predicting cancer driver mutations. Genome Biol 2020;21:43. 32079540 10.1186/s13059-020-01954-z PMC7033911

[vbag068-B14] Chen RJ , Lu MY, Williamson DFK et al Pan-cancer integrative histology-genomic analysis via multimodal deep learning. Cancer Cell 2022;40:865–78.e6. 35944502 10.1016/j.ccell.2022.07.004 PMC10397370

[vbag068-B15] Cheng J , Novati G, Pan J et al Accurate proteome-wide missense variant effect prediction with AlphaMissense. Science (New York, N.Y.) 2023;381:eadg7492. 37733863 10.1126/science.adg7492

[vbag068-B16] David A , Sternberg MJ. The contribution of missense mutations in core and rim residues of Protein-Protein interfaces to human disease. J Mol Biol 2015;427:2886–98. 26173036 10.1016/j.jmb.2015.07.004 PMC4548493

[vbag068-B17] Dildar M , Akram S, Irfan M et al Skin cancer detection: a review using deep learning techniques. IJERPH 2021;18:5479. 34065430 10.3390/ijerph18105479 PMC8160886

[vbag068-B18] Ding L , Getz G, Wheeler DA et al Somatic mutations affect key pathways in lung adenocarcinoma. Nature 2008;455:1069–75. 18948947 10.1038/nature07423 PMC2694412

[vbag068-B19] Engel J , Becker C, Lategahn J et al Insight into the inhibition of drug-resistant mutants of the receptor tyrosine kinase EGFR. Angew Chem Int Ed Engl 2016;55:10909–12. 27496389 10.1002/anie.201605011

[vbag068-B20] Erdős G , Dosztányi Z. Analyzing protein disorder with IUPred2A. Curr Protoc Bioinformatics 2020;70:e99. 32237272 10.1002/cpbi.99

[vbag068-B21] Esteva A , Kuprel B, Novoa RA et al Dermatologist-level classification of skin cancer with deep neural networks. Nature 2017;542:115–8. 28117445 10.1038/nature21056 PMC8382232

[vbag068-B22] Fernandez-Escamilla AM , Rousseau F, Schymkowitz J et al Prediction of sequence-dependent and mutational effects on the aggregation of peptides and proteins. Nat Biotechnol 2004;22:1302–6. 15361882 10.1038/nbt1012

[vbag068-B23] Gromiha MM. A statistical model for predicting protein folding rates from amino acid sequence with structural class information. J Chem Inf Model 2005;45:494–501. 15807515 10.1021/ci049757q

[vbag068-B24] Halperin I , Glazer DS, Wu S et al The FEATURE framework for protein function annotation: modeling new functions, improving performance, and extending to novel applications. BMC Genomics 2008;9:S2. 10.1186/1471-2164-9-S2-S2 PMC2559884 18831785

[vbag068-B25] Iengar P. An analysis of substitution, deletion and insertion mutations in cancer genes. Nucleic Acids Res 2012;40:6401–13. 22492711 10.1093/nar/gks290 PMC3413105

[vbag068-B26] Jennings JL , Hudson TJ. International cancer genome consortium (ICGC). Cancer Res 2016;76:130.

[vbag068-B27] Jones S , Zhang X, Parsons DW et al Core signaling pathways in human pancreatic cancers revealed by global genomic analyses. Science (New York, N.Y.) 2008;321:1801–6. 18772397 10.1126/science.1164368 PMC2848990

[vbag068-B28] Jumper J , Evans R, Pritzel A et al Highly accurate protein structure prediction with AlphaFold. Nature 2021;596:583–9. 34265844 10.1038/s41586-021-03819-2 PMC8371605

[vbag068-B29] Kabsch W , Sander C. Dictionary of protein secondary structure: pattern recognition of hydrogen-bonded and geometrical features. Biopolymers 1983;22:2577–637. 6667333 10.1002/bip.360221211

[vbag068-B30] Kamburov A , Lawrence MS, Polak P et al Comprehensive assessment of cancer missense mutation clustering in protein structures. Proc Natl Acad Sci U S A 2015;112:E5486–95. 26392535 10.1073/pnas.1516373112 PMC4603469

[vbag068-B31] Kandoth C , McLellan MD, Vandin F et al Mutational landscape and significance across 12 major cancer types. Nature 2013;502:333–9. 24132290 10.1038/nature12634 PMC3927368

[vbag068-B32] Kawashima S , Kanehisa M. AAindex: amino acid index database. Nucleic Acids Res 2000;28:374. 10592278 10.1093/nar/28.1.374 PMC102411

[vbag068-B33] Kawashima S , Pokarowski P, Pokarowska M et al AAindex: amino acid index database, progress report 2008. Nucleic Acids Res 2008;36:D202–D205. 17998252 10.1093/nar/gkm998 PMC2238890

[vbag068-B34] Kim M , Kim Y, Yoo J et al Regularized speaker adaptation of KL-HMM for dysarthric speech recognition. IEEE Trans Neural Syst Rehabil Eng 2017;25:1581–91. 28320669 10.1109/TNSRE.2017.2681691 PMC5591083

[vbag068-B35] Ko CW , Huh J, Park JW. Deep learning program to predict protein functions based on sequence information. MethodsX 2022;9:101622. 35111575 10.1016/j.mex.2022.101622 PMC8790617

[vbag068-B36] Kumar P , Henikoff S, Ng PC. Predicting the effects of coding non-synonymous variants on protein function using the SIFT algorithm. Nat Protoc 2009;4:1073–81. 19561590 10.1038/nprot.2009.86

[vbag068-B37] Landrum MJ , Lee JM, Benson M et al ClinVar: improving access to variant interpretations and supporting evidence. Nucleic Acids Res 2018;46:D1062–D1067. 29165669 10.1093/nar/gkx1153 PMC5753237

[vbag068-B38] Liao Z , Pan G, Sun C et al Predicting subcellular location of protein with evolution information and sequence-based deep learning. BMC Bioinformatics 2021;22:515. 34686152 10.1186/s12859-021-04404-0 PMC8539821

[vbag068-B39] Lin BC , Katneni U, Jankowska KI et al In silico methods for predicting functional synonymous variants. Genome Biol 2023;24:126. 37217943 10.1186/s13059-023-02966-1 PMC10204308

[vbag068-B40] Liu X , Li C, Mou C et al dbNSFP v4: a comprehensive database of transcript-specific functional predictions and annotations for human nonsynonymous and splice-site SNVs. Genome Med 2020;12:103. 33261662 10.1186/s13073-020-00803-9 PMC7709417

[vbag068-B41] Liu J , Zhao H, Zheng Y et al DrABC: deep learning accurately predicts germline pathogenic mutation status in breast cancer patients based on phenotype data. Genome Med 2022;14:21. 35209950 10.1186/s13073-022-01027-9 PMC8876403

[vbag068-B42] Luo P , Ding Y, Lei X et al deepDriver: predicting cancer driver genes based on somatic mutations using deep convolutional neural networks. Front Genet 2019;10:13. 30761181 10.3389/fgene.2019.00013 PMC6361806

[vbag068-B43] Mao Y , Chen H, Liang H et al CanDrA: cancer-specific driver missense mutation annotation with optimized features. PLoS One 2013;8:e77945. 24205039 10.1371/journal.pone.0077945 PMC3813554

[vbag068-B44] Mikhail DY , Al-Mukhtar FH, Kareem SW. A comparative evaluation of cancer classification via TP53 gene mutations using machin learning. Asian Pac J Cancer Prev 2022;23:2459–67. 35901354 10.31557/APJCP.2022.23.7.2459 PMC9727340

[vbag068-B45] Nelakurti DD , Rossetti T, Husbands AY et al Arginine depletion in human cancers. Cancers (Basel) 2021;13:6274. 34944895 10.3390/cancers13246274 PMC8699593

[vbag068-B46] Nourbakhsh M , Degn K, Saksager A et al Prediction of cancer driver genes and mutations: the potential of integrative computational frameworks. Brief Bioinform 2024;25:bbad519. 38261338 10.1093/bib/bbad519 PMC10805075

[vbag068-B47] Oh JH , Sung CO. Comprehensive characteristics of somatic mutations in the normal tissues of patients with cancer and existence of somatic mutant clones linked to cancer development. J Med Genet 2021;58:433–41. 32719100 10.1136/jmedgenet-2020-106905

[vbag068-B48] Pain M , Wang H, Lee E et al Treatment-associated TP53 DNA-binding domain missense mutations in the pathogenesis of secondary gliosarcoma. Oncotarget 2018;9:2603–21. 29416795 10.18632/oncotarget.23517 PMC5788663

[vbag068-B49] Pan X , Chen L, Liu M et al Identifying protein subcellular locations with embeddings-based node2loc. IEEE/ACM Trans Comput Biol Bioinform 2022;19:666–75. 33989156 10.1109/TCBB.2021.3080386

[vbag068-B50] Pandey M , Gromiha MM. Predicting potential residues associated with lung cancer using deep neural network. Mutat Res 2021;822:111737. 33508631 10.1016/j.mrfmmm.2020.111737

[vbag068-B51] Pandey M , Gromiha MM. MutBLESS: a tool to identify disease-prone sites in cancer using deep learning. Biochim Biophys Acta Mol Basis Dis 2023;1869:166721. 37105446 10.1016/j.bbadis.2023.166721

[vbag068-B52] Pandey M , Anoosha P, Yesudhas D et al Identification of potential driver mutations in glioblastoma using machine learning. Brief Bioinform 2022;23:bbac451. 36266243 10.1093/bib/bbac451

[vbag068-B53] Pejaver V , Urresti J, Lugo-Martinez J et al Inferring the molecular and phenotypic impact of amino acid variants with MutPred2. Nat Commun 2020;11:5918. 33219223 10.1038/s41467-020-19669-x PMC7680112

[vbag068-B54] Petukh M , Kucukkal TG, Alexov E. On human disease-causing amino acid variants: statistical study of sequence and structural patterns. Hum Mutat 2015;36:524–34. 25689729 10.1002/humu.22770 PMC4409542

[vbag068-B55] Quang D , Chen Y, Xie X. DANN: a deep learning approach for annotating the pathogenicity of genetic variants. Bioinformatics 2015;31:761–3. 25338716 10.1093/bioinformatics/btu703 PMC4341060

[vbag068-B56] Raimondi D , Passemiers A, Fariselli P et al Current cancer driver variant predictors learn to recognize driver genes instead of functional variants. BMC Biol 2021;19:3. 33441128 10.1186/s12915-020-00930-0 PMC7807764

[vbag068-B57] Rentzsch P , Witten D, Cooper GM et al CADD: predicting the deleteriousness of variants throughout the human genome. Nucleic Acids Res 2019;47:D886–D894. 30371827 10.1093/nar/gky1016 PMC6323892

[vbag068-B58] Rogers MF , Gaunt TR, Campbell C. CScape-somatic: distinguishing driver and passenger point mutations in the cancer genome. Bioinformatics 2020;36:3637–44. 32282885 10.1093/bioinformatics/btaa242 PMC7320610

[vbag068-B59] Schmidt A , Röner S, Mai K et al Predicting the pathogenicity of missense variants using features derived from AlphaFold2. Bioinformatics 2023;39:btad280. 37084271 10.1093/bioinformatics/btad280 PMC10203375

[vbag068-B60] Selvam K , Sivapragasam S, Poon GMK et al Detecting recurrent passenger mutations in melanoma by targeted UV damage sequencing. Nat Commun 2023;14:2702. 37169747 10.1038/s41467-023-38265-3 PMC10175485

[vbag068-B61] Sherry ST , Ward MH, Kholodov M et al dbSNP: the NCBI database of genetic variation. Nucleic Acids Res 2001;29:308–11. 11125122 10.1093/nar/29.1.308 PMC29783

[vbag068-B62] Siegel RL , Miller KD, Wagle NS et al Cancer statistics, 2023. CA Cancer J Clin 2023;73:17–48. 36633525 10.3322/caac.21763

[vbag068-B63] Sinkala M. Mutational landscape of cancer-driver genes across human cancers. Sci Rep 2023;13:12742. 37550388 10.1038/s41598-023-39608-2 PMC10406856

[vbag068-B64] Tan KP , Kanitkar TR, Kwoh CK et al Packpred: predicting the functional effect of missense mutations. Front Mol Biosci 2021;8:646288. 34490344 10.3389/fmolb.2021.646288 PMC8417552

[vbag068-B65] Tang YY , Wei PJ, Zhao JP et al Identification of driver genes based on gene mutational effects and network centrality. BMC Bioinformatics 2021;22:457. 34560840 10.1186/s12859-021-04377-0 PMC8461858

[vbag068-B66] Tate JG , Bamford S, Jubb HC et al COSMIC: the Catalogue of Somatic Mutations In Cancer. Nucleic Acids Res 2019;47:D941–D947. 30371878 10.1093/nar/gky1015 PMC6323903

[vbag068-B67] Thomas PD , Kejariwal A, Guo N et al Applications for protein sequence-function evolution data: mRNA/protein expression analysis and coding SNP scoring tools. Nucleic Acids Res 2006;34:W645–650. 16912992 10.1093/nar/gkl229 PMC1538848

[vbag068-B68] Tokheim C , Karchin R. CHASMplus reveals the scope of somatic missense mutations driving human cancers. Cell Syst 2019;9:9–23.e8. 31202631 10.1016/j.cels.2019.05.005 PMC6857794

[vbag068-B69] Troshin PV , Procter JB, Sherstnev A et al JABAWS 2.2 distributed web services for bioinformatics: protein disorder, conservation and RNA secondary structure. Bioinformatics 2018;34:1939–40. 29390042 10.1093/bioinformatics/bty045 PMC5972556

[vbag068-B70] Uzair M , Jamil N. Effects of hidden layers on the efficiency of neural networks. In: *2020 IEEE 23rd International Multitopic Conference (INMIC)*, IEEE, Bahawalpur, Pakistan, 2020.

[vbag068-B71] Valdar WS. Scoring residue conservation. Proteins 2002;48:227–41. 12112692 10.1002/prot.10146

[vbag068-B72] Vitkup D , Sander C, Church GM. The amino-acid mutational spectrum of human genetic disease. Genome Biol 2003;4:R72. 14611658 10.1186/gb-2003-4-11-r72 PMC329120

[vbag068-B73] Vogelstein B , Papadopoulos N, Velculescu VE et al Cancer genome landscapes. Science (New York, N.Y.) 2013;339:1546–58. 23539594 10.1126/science.1235122 PMC3749880

[vbag068-B74] Vyatkin AD , Otnyukov DV, Leonov SV et al Comprehensive patient-level classification and quantification of driver events in TCGA PanCanAtlas cohorts. PLoS Genet 2022;18:e1009996. 35030162 10.1371/journal.pgen.1009996 PMC8759692

[vbag068-B75] Wang H , Wang T, Zhao X et al AI-Driver: an ensemble method for identifying driver mutations in personal cancer genomes. NAR Genom Bioinform 2020;2:lqaa084. 33575629 10.1093/nargab/lqaa084 PMC7671397

[vbag068-B76] Wang S , Shi J, Ye Z et al Predicting EGFR mutation status in lung adenocarcinoma on computed tomography image using deep learning. Eur Respir J 2019;53:1800986. 30635290 10.1183/13993003.00986-2018 PMC6437603

[vbag068-B77] Weinstein JN , Collisson EA, Mills GB et al The Cancer Genome Atlas Pan-Cancer analysis project. Nat Genet 2013;45:1113–20. 24071849 10.1038/ng.2764 PMC3919969

[vbag068-B78] Wen G , Li H, Huang J et al Random deep belief networks for recognizing emotions from speech signals. Comput Intell Neurosci 2017;2017:1945630. 1945630. 28356908 10.1155/2017/1945630 PMC5357547

[vbag068-B79] Woldmar N , Schwendenwein A, Kuras M et al Proteomic analysis of brain metastatic lung adenocarcinoma reveals intertumoral heterogeneity and specific alterations associated with the timing of brain metastases. ESMO Open 2023;8:100741. 36527824 10.1016/j.esmoop.2022.100741 PMC10024110

[vbag068-B80] Yu HA , Suzawa K, Jordan E et al Concurrent alterations in EGFR-Mutant lung cancers associated with resistance to EGFR kinase inhibitors and characterization of MTOR as a mediator of resistance. Clin Cancer Res 2018;24:3108–18. 29530932 10.1158/1078-0432.CCR-17-2961 PMC6420806

[vbag068-B81] Yue Y , Huang Q, Zhu P et al Identification of pathogenic mutations and investigation of the NOTCH pathway activation in kartagener syndrome. Front Genet 2019;10:749. 31507630 10.3389/fgene.2019.00749 PMC6713718

[vbag068-B82] Yue Z , Zhao L, Xia J. dbCPM: a manually curated database for exploring the cancer passenger mutations. Brief Bioinform 2020;21:309–17. 30379998 10.1093/bib/bby105

[vbag068-B83] Zhou Y , Pan Q, Pires DEV et al DDMut: predicting effects of mutations on protein stability using deep learning. Nucleic Acids Res 2023;51:W122–W128. 37283042 10.1093/nar/gkad472 PMC10320186

