# Supplementary material for: Classification of driver and passenger mutations in different cancer types using deep neural networks
Source: Bioinform Adv. 2026 Feb 26;6(1):vbag068. doi: 10.1093/bioadv/vbag068 (PMC12989160; doi:10.1093/bioadv/vbag068)
Supplement: vbag068_Supplementary_Data [file vbag068_supplementary_data.docx]

**Supplementary Information**

**Table S1:**

**Preprocessed mutation data and description of cancer types along with their regions in human body**

| **Cancer subtypes** | **Groups** | **Cancer census genes (COSMIC CGC)** | | **Dataset after reducing redundancy** | | |
| --- | --- | --- | --- | --- | --- | --- |
|  |  | **Mutations** | **Proteins** | **Drivers** | **Passengers** | **Proteins** |
| Thymoma (THYM) | Hem Lymph | 135 | 37 | 3150 | 3139 | 293 |
| Acute myeloid leukemia (LAML) |  | 7523 | 473 |  |  |  |
| Lymphoid Neoplasm Diffuse Large B-cell Lymphoma (DLBC) |  | 8510 | 500 |  |  |  |
| Breast carcinoma (BRCA) | Breast | 33065 | 695 | 5577 | 3553 | 529 |
| Colorectal adenocarcinoma (COADREAD/READ*) | COADREAD | 91022 | 707 | 13953 | 3735 | 587 |
| Stomach adenocarcinoma (STAD) | STAD | 32959 | 702 | 4217 | 2624 | 529 |
| Esophageal carcinoma (ESCA) | Digestive | 14455 | 663 | 2860 | 2672 | 386 |
| Cholangiocarcinoma (CHOL) |  | 226 | 88 |  |  |  |
| Liver hepatocellular carcinoma (LIHC) |  | 10481 | 652 |  |  |  |
| Pancreatic adenocarcinoma (PAAD) |  | 11333 | 643 |  |  |  |
| Kidney chromophobe (KICH) | Kidney | 429 | 109 | 1749 | 1600 | 316 |
| Kidney renal clear cell carcinoma (KIRC) |  | 9246 | 638 |  |  |  |
| Kidney renal papillary cell carcinoma (KIRP) |  | 2724 | 491 |  |  |  |
| Bladder urothelial carcinoma (BLCA) | BLCA | 25610 | 696 | 3785 | 2994 | 418 |
| Lung adenocarcinoma (LUAD) | LUAD | 44162 | 704 | 5788 | 2995 | 419 |
| Lung squamous cell carcinoma (LUSC) | Thoracic | 23583 | 695 | 3025 | 2982 | 336 |
| Mesothelioma (MESO) |  | 1139 | 267 |  |  |  |
| Ovarian serous cystadenocarcinoma (OV) | Gynecologic | 10996 | 642 | 7373 | 3193 | 457 |
| Cervical squamous cell carcinoma and endocervical adenocarcinoma (CESC) |  | 6479 | 615 |  |  |  |
| Uterine carcinosarcoma (UCS) |  | 53 | 28 |  |  |  |
| Uterine corpus endometrial carcinoma (UCEC) |  | 41804 | 705 |  |  |  |
| Adrenocortical carcinoma (ACC) | Endocrine | 897 | 186 | 2668 | 2718 | 428 |
| Thyroid carcinoma (THCA) |  | 10930 | 656 |  |  |  |
| Pheochromocytoma and Paraganglioma (PCPG) |  | 250 | 39 |  |  |  |
| Skin cutaneous melanoma (SKCM) | Skin | 70041 | 706 | 9939 | 3563 | 552 |
| Head and neck squamous cell carcinoma (HNSC) | HNSC | 19192 | 688 | 3168 | 2415 | 344 |
| Sarcoma (SARC) | Others | 10006 | 624 | 4709 | 3496 | 521 |
| Uveal Melanoma (UVM) |  | 154 | 17 |  |  |  |
| Testicular Germ Cell Tumors (TGCT) |  | 11 | 2 |  |  |  |
| Prostate adenocarcinoma (PRAD) |  | 8952 | 645 |  |  |  |

*combined COADREAD and READ cancer types because individual information was not available

*LGG and GBM are merged with keywords glioma and glioblastoma

**Table S2:** Proteins with ≥50 mutations in at least three cancer types

| **UniProt ID** | **Gene Name** | **Number of driver mutations** | **UniProt ID** | **Gene Name** | **Number of driver mutations** |
| --- | --- | --- | --- | --- | --- |
| **P04637** | **P53** | 219 | P20823 | HNF1A | 57 |
| **P23468** | **PTPRD** | 104 | P21802 | FGFR2 | 56 |
| **P35222** | **CTNB1** | 95 | **P38398** | **BRCA1** | 55 |
| P26232 | CTNA2 | 92 | Q86U86 | PB1 | 55 |
| O14522 | PTPRT | 86 | P49815 | TSC2 | 55 |
| Q12879 | NMDE1 | 82 | Q8IXJ9 | ASXL1 | 54 |
| Q9NYF8 | BCLF1 | 81 | P04629 | NTRK1 | 54 |
| P10415 | BCL2 | 74 | P35240 | MERL | 54 |
| P35916 | VGFR3 | 73 | O14746 | TERT | 53 |
| Q9H3D4 | P63 | 73 | P12830 | CADH1 | 53 |
| P51532 | SMCA4 | 69 | **P00533** | **EGFR** | **53** |
| Q16288 | NTRK3 | 69 | Q13485 | SMAD4 | 53 |
| P10275 | ANDR | 69 | Q02548 | PAX5 | 53 |
| Q68DV7 | RNF43 | 69 | Q9UER7 | DAXX | 53 |
| Q9Y2T1 | AXIN2 | 69 | Q969V6 | MRTFA | 53 |
| P28340 | DPOD1 | 66 | Q969H0 | FBXW7 | 52 |
| P29320 | EPHA3 | 65 | Q9HBE5 | IL21R | 52 |
| P07949 | RET | 64 | P31749 | AKT1 | 52 |
| Q06455 | MTG8 | 61 | P08581 | MET | 52 |
| P05771 | KPCB | 59 | Q6W2J9 | BCOR | 51 |
| Q92574 | TSC1 | 58 | Q9NQB0 | TF7L2 | 51 |
| P52333 | JAK3 | 58 |  |  |  |

**Table S3:** Preference of driver mutations

|  | **A** | **C** | **D** | **E** | **F** | **G** | **H** | **I** | **K** | **L** | **M** | **N** | **P** | **Q** | **R** | **S** | **T** | **V** | **W** | **Y** |
| --- | --- | --- | --- | --- | --- | --- | --- | --- | --- | --- | --- | --- | --- | --- | --- | --- | --- | --- | --- | --- |
| **A** | 0 | - | 0.44 | 0.22 | 0.01 | 0.31 | - | - | - | - | - | - | 0.38 | - | - | 0.90 | 2.66 | 2.65 | - | - |
| **C** | - | 0 | - | - | 0.27 | 0.10 | - | - | - | - | - | - | - | - | 0.26 | 0.19 | - | - | 0.11 | 0.46 |
| **D** | 0.15 | 0.01 | 0 | 0.56 | - | 0.75 | 0.79 | 0.01 | - | - | - | 2.66 | - | - | - | - | - | 0.36 | - | 1.13 |
| **E** | 0.21 | - | 1.48 | 0 | - | 0.62 | - | - | 4.69 | - | - | - | - | 1.37 | - | - | - | 0.34 | - | - |
| **F** | - | 0.22 | - | - | 0 | - | - | 0.17 | - | 0.98 | - | - | - | - | - | 0.25 | - | 0.22 | - | 0.08 |
| **G** | 0.45 | 0.71 | 1.02 | 1.42 | 0.01 | 0 | - | 0.01 | 0.02 | 0.02 | 0.01 | 0.02 | - | - | 1.61 | 0.96 | - | 1.15 | 0.51 | - |
| **H** | - | - | 0.18 | - | - | - | 0 | - | - | 0.14 | - | 0.36 | 0.16 | 0.33 | 0.43 | - | - | - | - | 0.96 |
| **I** | - | - | - | - | 0.25 | - | - | 0 | 0.04 | 0.18 | 0.60 | 0.19 | - | - | 0.03 | 0.15 | 0.47 | 0.59 | - | - |
| **K** | - | - | - | 0.58 | - | - | - | 0.07 | 0 | - | 0.17 | 1.47 | - | 0.27 | 0.57 | - | 0.57 | - | - | - |
| **L** | 0.01 | - | - | - | 1.34 | - | 0.21 | 0.81 | - | 0 | 0.60 | - | 0.90 | 0.22 | 0.48 | 0.20 | - | 0.89 | 0.05 | - |
| **M** | - | - | - | - | - | - | - | 1.14 | 0.10 | 0.19 | 0 | - | - | - | 0.07 | - | 0.26 | 0.33 | - | - |
| **N** | - | - | 0.33 | - | 0.01 | - | 0.25 | 0.23 | 0.49 | - | - | 0 | - | - | - | 0.75 | 0.21 | - | - | 0.17 |
| **P** | 0.46 | - | - | - | 0.07 | - | 0.93 | - | 0.01 | 2.85 | - | 0.01 | 0 | 0.54 | 0.37 | 2.86 | 0.88 | - | - | - |
| **Q** | - | - | - | 0.54 | - | - | 1.12 | - | 0.67 | 0.31 | - | - | 0.21 | 0 | 0.55 | - | - | - | - | - |
| **R** | - | 2.59 | - | - | - | 0.57 | 2.52 | 0.48 | 0.77 | 1.02 | 0.28 | - | 0.36 | 2.83 | 0 | 0.70 | 0.38 | - | 1.88 | - |
| **S** | 0.20 | 0.81 | - | - | 2.06 | 0.38 | - | 0.40 | - | 1.72 | - | 0.84 | 0.55 | - | 0.53 | 0 | 0.37 | - | 0.05 | 0.73 |
| **T** | 0.91 | - | - | - | 0.01 | - | - | 0.99 | 0.24 | - | 0.94 | 0.29 | 0.30 | - | 0.14 | 0.47 | 0 | - | - | - |
| **V** | 0.87 | - | 0.13 | 0.14 | 0.33 | 0.32 | - | 1.13 | - | 0.79 | 0.98 | - | - | - | - | 0.01 | - | 0 | - | - |
| **W** | - | 0.31 | - | - | - | 0.05 | - | - | - | 0.23 | - | - | - | - | 0.19 | 0.04 | - | - | 0 | - |
| **Y** | - | 0.72 | 0.11 | - | 0.21 | - | 0.46 | - | - | - | - | 0.17 | - | - | - | 0.11 | - | - | - | 0 |

Top 25 substitutions present in the driver mutations are highlighted in red shade

**Table S4:** Preference of passenger mutations

|  | **A** | **C** | **D** | **E** | **F** | **G** | **H** | **I** | **K** | **L** | **M** | **N** | **P** | **Q** | **R** | **S** | **T** | **V** | **W** | **Y** |
| --- | --- | --- | --- | --- | --- | --- | --- | --- | --- | --- | --- | --- | --- | --- | --- | --- | --- | --- | --- | --- |
| **A** | 0 | - | 0.29 | 0.16 | - | 0.57 | - | - | - | 0.03 | - | - | 0.65 | - | - | 1.10 | 3.11 | 2.09 | - | - |
| **C** | - | 0 | - | - | 0.05 | 0.03 | - | - | - | - | - | - | - | - | 0.24 | 0.24 | - | - | 0.18 | 0.26 |
| **D** | 0.18 | - | 0 | 2.01 | - | 0.76 | 0.68 | - | - | - | - | 1.51 | - | - | - | - | - | 0.29 | - | 0.68 |
| **E** | 0.39 | - | 2.01 | 0 | - | 0.50 | - | - | 1.62 | - | - | 0.03 | - | 0.63 | - | - | - | 0.26 | - | - |
| **F** | - | 0.08 | 0.03 | - | 0 | - | - | 0.10 | - | 1.04 | - | - | - | - | - | 0.16 | - | 0.03 | - | 0.03 |
| **G** | 0.55 | 0.31 | 0.63 | 0.60 | - | 0 | - | - | - | - | - | - | - | - | 1.10 | 1.38 | - | 0.63 | 0.16 | - |
| **H** | - | - | 0.16 | - | - | - | 0 | - | - | 0.42 | - | 0.24 | 0.13 | 0.91 | 0.89 | - | - | - | - | 0.52 |
| **I** | - | - | - | - | 0.13 | - | - | 0 | 0.05 | 0.65 | 1.28 | 0.13 | - | - | - | 0.13 | 0.73 | 2.22 | - | - |
| **K** | 0.05 | - | - | 1.10 | - | - | - | 0.10 | 0 | - | 0.10 | 1.04 | - | 0.21 | 1.10 | - | 0.31 | - | - | - |
| **L** | - | - | - | - | 1.25 | - | 0.13 | 0.47 | - | 0 | 0.37 | - | 0.57 | 0.05 | 0.26 | 0.26 | - | 1.33 | 0.05 | - |
| **M** | - | - | - | - | - | - | - | 0.99 | 0.13 | 0.24 | 0 | - | - | - | 0.05 | - | 0.76 | 0.99 | - | - |
| **N** | - | - | 0.68 | - | - | - | 0.31 | 0.57 | 1.49 | - | - | 0 | - | - | - | 2.01 | 0.29 | - | - | 0.21 |
| **P** | 0.71 | - | - | - | - | - | 0.29 | - | 0.03 | 2.48 | - | 0.03 | 0 | 0.39 | 0.71 | 2.61 | 1.15 | - | - | - |
| **Q** | - | - | - | 0.47 | - | - | 1.36 | - | 0.21 | 0.37 | - | - | 0.34 | 0 | 0.84 | - | - | - | - | - |
| **R** | 0.03 | 1.20 | - | - | - | 0.97 | 2.01 | 0.08 | 0.71 | 0.76 | 0.08 | - | 0.57 | 2.19 | 0 | 0.71 | 0.10 | - | 0.99 | - |
| **S** | 0.57 | 0.76 | 0.03 | - | 0.65 | 0.94 | - | 0.31 | - | 1.12 | - | 1.12 | 0.81 | - | 1.57 | 0 | 0.71 | - | 0.05 | 0.26 |
| **T** | 1.67 | - | - | - | - | - | - | 1.36 | 0.34 | - | 1.31 | 0.26 | 0.63 | - | 0.24 | 0.86 | 0 | - | - | - |
| **V** | 0.94 | - | 0.13 | 0.13 | 0.42 | 0.37 | - | 2.27 | - | 1.23 | 1.51 | - | - | - | - | - | - | 0 | - | - |
| **W** | - | 0.10 | - | - | - | - | - | - | - | 0.13 | - | - | 0.03 | - | 0.10 | 0.03 | - | 0.03 | 0 | - |
| **Y** | 0.03 | 0.55 | 0.03 | - | 0.18 | - | 0.31 | - | - | - | - | 0.05 | - | - | - | 0.08 | - | - | - | 0 |

Top 25 substitutions present in the passenger mutations are highlighted in green shade

**Table S5:** Odds ratio between driver and passenger mutations

|  | **A** | **C** | **D** | **E** | **F** | **G** | **H** | **I** | **K** | **L** | **M** | **N** | **P** | **Q** | **R** | **S** | **T** | **V** | **W** | **Y** |
| --- | --- | --- | --- | --- | --- | --- | --- | --- | --- | --- | --- | --- | --- | --- | --- | --- | --- | --- | --- | --- |
| **A** | 0 | - | 1.52 | 1.42 |  | 0.55 | - |  |  | 0 | - |  | 0.59 | - | - | 0.82 | 0.86 | 1.27 | - |  |
| **C** |  | 0 | - | - | 5.09 | 3.73 | - | - | - |  | - | - |  | - | 1.1 | 0.81 |  |  | 0.6 | 1.76 |
| **D** | 0.85 |  | 0 | 0.28 |  | 0.99 | 1.16 |  |  | - | - | 1.76 | - | - |  |  | - | 1.25 | - | 1.66 |
| **E** | 0.53 | - | 0.74 | 0 | - | 1.24 | - |  | 2.9 |  | - | 0.07 | - | 2.19 | - |  | - | 1.31 | - |  |
| **F** | - | 2.8 | 0 | - | 0 |  | - | 1.581 |  | 0.94 | - | - |  | - | - | 1.58 | - | 8.25 | - | 3.06 |
| **G** | 0.82 | 2.3 | 1.62 | 2.36 |  | 0 |  |  |  |  |  |  |  | - | 1.5 | 0.69 |  | 1.83 | 3.3 |  |
| **H** | - | - | 1.14 | - |  | - | 0 | - | - | 0.34 | - | 1.53 | 1.21 | 0.36 | 0.5 | - | - | - | - | 1.84 |
| **I** | - | - | - | - | 1.92 | - |  | 0 | 0.77 | 0.27 | 0.47 | 1.49 | - | - |  | 1.14 | 0.64 | 0.27 | - | - |
| **K** | 0 | - |  | 0.53 | - | - | - | 0.649 | 0 |  | 1.597 | 1.4 | - | 1.3 | 0.5 | - | 1.81 | - |  |  |
| **L** |  | - | - | - | 1.07 |  | 1.62 | 1.723 | - | 0 | 1.64 | - | 1.56 | 4.19 | 1.8 | 0.76 | - | 0.67 | 1 | - |
| **M** | - | - | - |  | - | - | - | 1.145 | 0.8 | 0.79 | 0 |  | - | - | 1.3 | - | 0.35 | 0.33 | - | - |
| **N** | - | - | 0.49 | - |  | - | 0.8 | 0.408 | 0.33 |  |  | 0 | - |  | - | 0.38 | 0.74 | - | - | 0.8 |
| **P** | 0.65 | - |  | - |  | - | 3.23 | - | 0.2 | 1.15 | - | 0.27 | 0 | 1.38 | 0.5 | 1.09 | 0.77 |  | - | - |
| **Q** |  | - | - | 1.16 | - | - | 0.83 | - | 3.19 | 0.84 | - | - | 0.62 | 0 | 0.7 | - |  |  | - | - |
| **R** | 0 | 2.2 | - | - |  | 0.59 | 1.25 | 6.1 | 1.09 | 1.34 | 3.616 |  | 0.62 | 1.29 | 0 | 0.99 | 3.59 | - | 1.9 |  |
| **S** | 0.34 | 1.1 | 0.07 | - | 3.16 | 0.4 | - | 1.276 |  | 1.53 | - | 0.75 | 0.68 | - | 0.3 | 0 | 0.53 |  | 1 | 2.78 |
| **T** | 0.55 | - | - | - |  | - |  | 0.727 | 0.7 | - | 0.723 | 1.12 | 0.48 |  | 0.6 | 0.55 | 0 | - | - | - |
| **V** | 0.93 | - | 1.02 | 1.09 | 0.79 | 0.87 | - | 0.5 |  | 0.65 | 0.644 |  | - |  |  |  | - | 0 |  | - |
| **W** | - | 3 | - | - |  |  | - | - | - | 1.78 | - | - | 0 | - | 1.8 | 1.6 | - | 0 | 0 | - |
| **Y** | 0 | 1.3 | 4.33 | - | 1.14 | - | 1.46 | - | - | - | - | 3.23 | - |  |  | 1.46 | - | - | - | 0 |

Top 25 substitutions preferred in driver and passengers are represented in red and green shades, respectively. Yellow shaded substitutions are present in only drivers.

**Table S6:** Top ten mutations in highly mutated genes in different cancer types

| **Group** | **1** | **2** | **3** | **4** | **5** | **6** | **7** | **8** | **9** | **10** |
| --- | --- | --- | --- | --- | --- | --- | --- | --- | --- | --- |
| **Hem.Lymph** | BCL2  **(A→V)** | SOCS1  (L**→**V) | DNM3A  (C**→**Y) | TET2  (P**→**L) | PIM1  (L**→**V) | SGK1  (A**→**G) | RUNX1  (D**→**Y) | ASXL1  **(A→T)** | MYC  (S**→**N) | GATA2  **(R→Q)** |
| **BRCA** | **PTPRD**  **(E→K)** | KMT2C  **(E→K)** | **P53**  (P**→**L) | CADH1  (P**→**S) | NMDE1  **(A→T)** | ATM  **(E→K)** | BRCA1  **(E→K)** | CTCF  **(E→K)** | ESR1  (G**→**D) | STAG2  **(E→K)** |
| **COADREAD** | FBXW7  (T**→**I) | SMAD4  **(A→V)** | **PTPRD**  **(R→C)** | TF7L2  (P**→**L) | **CTNB1**  **(A→T)** | VGFR3  **(A→T)** | DICER  (R**→**H) | **SMCA4**  (R**→**H) | FLNA  **(A→V)** | NMDE1  **(A**→**T)** |
| **STAD** | CADH1  (L**→**P) | **PTPRD**  (K**→**T) | KAT6A  **(R→Q)** | **SMCA4**  (R**→**H) | NMDE1  (M**→**I) | CTNA2 (K**→**N) | VGFR3  (L**→**M) | TENA  **(E→K)** | NUMA1  **(A→T)** | NIN  (E**→**D) |
| **Digestive** | SMAD4  (D**→**A) | MEN1  **(A→V)** | **PTPRD**  (N**→**S) | **CTNB1**  **(****D→N)** | RNF43  (C**→**Y) | **P53**  (G**→**A) | HNF1A  (P**→**L) | NBEA  (E**→**D) | **SMCA4**  **(A→V)** | TSC2  (R**→**W) |
| **Kidney** | VHL  (L**→**P) | PB1  (Y**→**C) | **SMCA4**  **(R→Q)** | KAT6A  (D**→**G) | **PTPRD**  (P**→**L) | MTOR  (A**→**P) | ZMYM2  **(A→T)** | VGFR3  (H**→**Y) | TSC2  (F**→**I) | KDM5C  (E**→**Q) |
| **BLCA** | **P53**  (P**→**L) | STAG2  (D**→**H) | **PTPRD**  **(E→K)** | TSC1  (P**→**L) | PB1  **(E→K)** | **SMCA4**  **(E→K)** | BRCA1  **(E→K)** | DICER  (Q**→**E) | ELF3  (K**→**N) | ASXL1  **(D→N)** |
| **LUAD** | **PTPRD**  (D**→**Y) | NMDE1  (D**→**Y) | NTRK3  (G**→**V) | EGFR  (V**→**M) | EPHA3  (P**→**Q) | **SMCA4**  (G**→**V) | PTPRT  (G**→**V) | CTNA2  (A**→**S) | BCLF1  (S**→**Y) | VGFR3  (A**→**E) |
| **Thoracic** | CTNA2  (A**→**D) | **PTPRD**  (R**→**L) | MTG8  (A**→**S) | NMDE1  (A**→**S) | BCLF1  (E**→**D) | ASXL1  **(D→N)** | DICER  **(E→K)** | RGS7  (D**→**Y) | NTRK3  (A**→**P) | EPHA3  (A**→**P) |
| **Gynecologic** | **PTPRD**  (E**→**Q) | CHD4  (D**→**H) | FLNA  **(D→N)** | TSC2  (L**→**F) | BRCA1  (K**→**R) | **SMCA4**  **(E→K)** | ASXL1  (G**→**S) | NTRK3  (D**→**E) | DICER  **(A→V)** | MYOME  (Q**→**H) |
| **Endocrine** | **MUC4**  (V**→**A) | RET  (C**→**R) | **CTNB1**  (K**→**R) | **SMCA4**  (L**→**P) | NF1  (A**→**V) | MYO5A  (K**→**N) | PB1  **(E→K)** | SET1B  (L**→**P) | MEN1  (V**→**D) | MED12  (P**→**H) |
| **SKCM** | NMDE1  **(E→K)** | **PTPRD**  **(E→K)** | MUC16  (S**→F)** | PTPRT  **(E→K)** | BCLF1  **(E→K)** | NBEA  **(E→K)** | NTRK3  **(E→K)** | PTPRK  P**→**S) | TET2  (P**→**L) | P63  **(E→K)** |
| **HNSC** | **MUC4**  **(A→T)** | **P53**  (G**→**E) | **PTPRD**  (S**→**R) | PABP1  (R**→**C) | MYOME  **(A→T)** | HLAA  (E**→**G) | NBEA  **(E→K)** | **SMCA4**  **(E→K)** | CASP8  (F**→**L) | CTNA2  (G**→**V) |
| **UCEC** | FBXW7  (V**→**L) | 2AAA  (W**→**C) | BRCA1  (S**→**N) | **CTNB1**  (R**→**H) | FANCJ  (S**→**A) | KSYK  (Y**→**C) | MED12  (D**→**Y) | **P53**  (I**→**V) | RASK  (I**→**N) |  |
| **Others** | **PTPRD**  **(D→N)** | **P53**  (E**→**V) | **MUC4**  (P**→**L) | CHK2  (R**→**C) | NMDE1  (Q**→**K) | CTNA2  (G**→**R) | NIN  (E**→**Q) | ANDR  (P**→**S) | DICER  (I**→**V) | **SMCA4**  (P**→**L) |

| **Cancer type** | **Driver** | | | | | | |
| --- | --- | --- | --- | --- | --- | --- | --- |
|  | **XM** | **X*M** | **X**M** | **XMX** | **MX** | **M*X** | **M**X** |
| **Heme Lymph** | C**R**,R**Y**,N**Y**,F**G**,F**A** | E***I**,N***I**,Q***V**,A***E**,Q***M** | G****F**,*R******F***,G****Y**,C****S**,T****Q** | S**R**L,*T****P****P*,S**G**I,P**P**V,G**G**V | **Y**E,**E**K,**G**P,**Y**I,**K**Q | **G***V,**A***R,**H***G,**C***V,**C***G | **Y****M,F******R,**F****S,**Y****S,**K****P |
| **BRCA** | M**E**,F**L**,*S****C***,F**E** | G***E**,*T*****E***,C***G**,P***F**,N***F** | V****C**,N****C**,M****M**,T****Y**,C****A** | A**G**G,V**R**V,*T****P****P*,R**S**P,L**R**V | ***H****H*,***F****L*,**Y**E,**K**M,**M**F | **G***H,**H***K,**E***K,**S***M,**D***H | **G****N,**Q****T,**Q****K,***Q******Y*,**F****S |
| **COADREAD/READ** | T**C**,F**L**,V**K**,R**Y**,*S****C*** | C***G**,W***R**,*T*****E***,Y***F**,N***F** | V****C**,C****A**,M****R**,E****F**,H****R** | L**R**N,K**R**R,V**R**V,G**R**L,A**R**L | ***F****L*,**R**Y,***H****H*,**F**I,**R**L | **V***M,**A***W,**C***C,**C***F,**C***H | **I****M,**M****N,**H****V,**M****T,**I****T |
| **STAD** | V**K**,M**G**,M**D**,D**G**,M**R** | *T*****E***,R***E**,L***G**,Y***K**,Y***R** | M****R**,V****C**,V****A**,A****M**,N****C** | A**G**G,*T****P****P*,L**R**V,V**R**V,R**R**R | **L**M,**K**H,***H****H*,**C**Q,**K**Y | **V***M,**A***R,**V***F,**G***H,**F***Q | **Q****K,***Q******Y*,**G****F,**R****R,**Y****V |
| **Digestive** | *W****R***,*S****C***,Y**F**,Q**G**,W**V** | Y***Y**,N***F**,M***I**,Y***F**,V***G** | V****C**,*R******F***,N****F**,L****H**,H****G** | V**R**A,V**G**S,S**L**S,H**S**G,A**G**G | **R**Y,**H**Y,***F****L*,**A**Q,**C**T | **V***F,**V***C,**L***R,**Y***D,**H***K | **F****G,**V****W,***Q******Y*,**V****Y,**Q****K |
| **Kidney** | *R****C***,H**L**,V**Y**,V**W**,G**M** | R***H**,Y***G**,E***Y**,P***W**,H***W** | **L****W,**M****L,**H****G,**W****R,**V****C | V**R**S,T**R**L,*T****P****P*,R**S**V,Q**H**L | **W**L,**Q**V,**S**Y,**G**M,**F**Q | **C***V,**C***G,**L***R,**G***H,**V***F | **Y****I,**C****L,**P****F,**L****F,**G****N |
| **BLCA** | F**E**, A**E**, F**L,**V**K**, S**Q** | Y***E**,C***G**,*T*****E***,G***E**, R***H** | M****R**,W****E**,G****M**,V****C**,L****H** | A**G**G,D**E**L,N**S**S,L**E**V,T**P**P | **E**K,**R**Y,**E**R,**E**G,**C**Q | **E***Y,**Q***C,**L***R,**F***D,**E***K | **Q****K,***Q******Y*,**Q****T,**G****N,**G****F |
| **LUAD** | V**K**,*R****C***,*W****R***,*S****C***,F**L** | K***G**,I***Q**,*T*****E***,G***Q**,Y***E** | V****C**,N****C**,L****H**,Y****Q**,F****Q** | A**G**G,Q**H**L,*T****P****P*,T**G**L,S**S**G | **P**H,**R**Y,**Q**T,**C**Q,**D**F | **Q***Y,**Q***N,**Q***W,**L***R,**D***H | **Q****K,**G****F,***Q******Y*,**W****S,**Q****T |
| **Gynecologic** | F**L**,H**E**,F**E**,Y**E**,I**G** | W***R**,G***E**,Y***K**,Y***E**,Y***F** | M****R**,E****F**,C****G**,G****K**,V****C** | L**R**V,K**R**I,P**R**T,Q**R**S,N**S**S | **R**Y,**E**H,**E**K,**P**H,**Q**T | **L***R,**F***D,**E***K,**K***F,**D***H | **F****S,**F****K,**Q****T,**Q****K,**Y****E |
| **Endocrine** | Q**G**,G**Q**,F**G**,V**C**,F**E** | G***Q**,R***H**,W***Q**,H***P**,W***R** | M****M**,D****C**,Y****Q**,T****E**,Y****C** | A**G**G,S**A**S,S**V**S,T**R**L,L**H**V | **Q**T,**L**D,***H****H*,**G**P,**E**M | **G***H,**E***Y,**C***P,**Y***P,**H***E | ***Q******Y*,**G****N,**Q****K,**L****M,**H****P |
| **SKCM** | M**E**,F**L**,W**E**,F**P**,Y**P** | *T*****E***,C***G**,Y***G**,Y***E**,W***P** | W****G**,G****M**,H****G**,M****G**,V****G** | S**P**V,L**P**S,A**G**G,*T****P****P*,I**P**V | **S**Y,**P**W,**P**H,**G**I,**G**N | **E***K,**G***H,**S***M,**E***Y,**P***W | **P****M,**G****Y,**G****F,**Q****Y,**P****C |
| **HNSC** | I**I**,C**P**,*R****C***,H**A**,G**H** | *T*****E***,R***E**,C***V**,R***Q**,K***G** | M****R**,V****C**,T****Y**,N****C**,G****M** | L**P**V,S**L**S,S**V**S,A**G**G,S**A**S | ***H****H*,**E**M,**G**F,**H**T,**H**M | **G***V,**H***T,**K***F,**D***K,**E***K | **H****P,***Q******Y*,**Q****K,**M****P,**G****N |
| **Others** | T**Y**,F**L**,*S****C***,*W****R***,Y**M** | Y***F**,*T*****E***,G***Q**,S***Y**,W***P** | M****M**,H****G**,*R******F***,V****C**,N****F** | S**L**S,G**G**V,A**P**A,V**D**S,I**N**L | **F**K,***H****H*,**M**R,**Y**E,**Y**D | **G***W,**Q***Y,**R***W,**A***F,**Y***P | **G****N,**Q****K,**V****M,**Y****A,**T****C |

**Table S7a:** Preferred motifs in drivers across cancer types

| **Cancer type** | **Passenger** | | | | | | |
| --- | --- | --- | --- | --- | --- | --- | --- |
|  | **XM** | **X*M** | **X**M** | **XMX** | **MX** | **M*X** | **M**X** |
| **Heme Lymph** | S**M**,N**E**,D**M**,D**Q**,P**M** | E***Y**,T***E**,F***S**,W***R**,R***F** | D****K**,M****D**,M****P**,M****V**,R****M** | R**R**S,E**R**G,E**A**A,I**K**V,K**P**S | **N**D,**S**M,**M**E,**T**I,**K**A | **I***E,**V***N,**I***P,**K***Q,**M***L | **T****N,**V****F,**I****P,**Q****S,**S****T |
| **BRCA** | I**F**,H**T**,W**M**,Y**N**,T**N** | H***H**,M***Q**,T***Y**,Y***I**,W***V** | M****D**,Q****N**,W****A**,H****H**,H****K** | T**R**E,P**V**S,K**A**S,N**I**N,P**P**S | ***N****Q*,**F**H,**N**C,**Q**C,**M**V | **N***Q,**N***H,**M***V,**I***Q,**F***R | **N****Y,**N****K,**F****N,**H****M,**M****F |
| **COADREAD/READ** | C**I**,*W****M***,N**H**,Y**H**,D**M** | Y***H**,M***M**,F***N**,D***I**,E***M** | M****N**,N****I**,W****N**,E****M**,Q****N** | E**N**S,L**N**V,N**I**N,S**I**G,T**S**Q | **N**E,**M**D,**I**A,**H**K,**N**D | **I***Q,**N***C,**N***H,**N***P,**N***W | **V****M,**Q****K,**Q****Y,**Q****T,**G****Y |
| **STAD** | R**N**,H**I**,A**F**,*W****M***,T**N** | D***P**,I***N**,A***F**,N***H**,Q***V** | Q****M**,A****N**,D****M**,H****Q**,M****F** | P**V**T,E**A**A,G**G**T,G**S**S,K**P**S | **I**G,**P**F,**H**G,**I**A,**I**V | **H***S,**I***Q,**I***D,**H***H,**Q***D | **K****I,**Q****F,**Y****R,**N****S,**T****N |
| **Digestive** | T**N**,N**I**,D**N**,I**S**,P**T** | D***I**,Y***I**,N***N**,Q***M**,V***K** | *N******N***,A****N**,K****H**,M****P**,R****T** | P**R**P,A**T**S,L**R**A,P**V**T,S**P**A | **T**Q,**M**S,**T**H,**A**H,**I**G | **T***F,**N***E,**K***P,**Q***D,**S***F | **I****P,**H****V,**N****M,**F****P,**Q****S |
| **Kidney** | D**N**,A**I**,Q**P**,H**T**,I**V** | G***D**,P***D**,S***N**,F***P**,K***S** | D****T**,K****N**,E****N**,H****V**,K****D** | S**S**S,P**R**P,T**D**A,E**E**D,E**R**E | **V**S,**T**K,**S**N,**A**D,**A**G | **H***S,**V***G,**T***S,**A***C,**S***D | **N****K,**M****T,**T****G,**P****R,**P****I |
| **BLCA** | G**H**, Q**I**, R**P**, D**M**, K**H**, N**H** | D***N**,F***K**,S***N**,F***I**,F***P** | Q****N**,*N******N***,I****N**, I****N**,N****M** | E**A**A, P**V**S,K**E**A,P**P**S,A**R**Q | **I**I,***N****Q*, **N**I, **F**G,**K**D | **N***D,**N***T,**T***D,**I***Q,**T***V | **N****S,**I****P,**M****D,**N****A,**V****Q |
| **LUAD** | E**N**,C**I**,*W****M***,C**K**,H**N** | Y***I**,C***T**,C***I**,W***E**,S***N** | W****A**,T****I**,M****F**,E****N**,K****N** | *T****R****E*,E**A**A,E**N**S,E**R**E,E**R**G | **I**P,**T**K,**F**H,**N**K,***N****Q* | **Y***W,**N***E,**F***S,**I***Q,**I***R | **I****V,**I****T,**F****D,**T****H,**I****L |
| **Gynecologic** | *W****M***,Y**N**,S**I**,P**N**,V**M** | H***H**,F***I**,N***N**,D***N**,K***Q** | Q****M**,P****H**,M****D**,R****Q**,V****H** | *T****R****E*,T**V**D,P**S**P,A**Q**A,A**R**D | **Q**F,**C**E,**C**G,**I**D,**H**G | **N***C,**I***Q,**Q***D,**N***W,**M***Q | ***I******M*,**H****K,**H****A,**N****D,**I****S |
| **Endocrine** | N**D**,H**I**,R**I**,D**I**,D**M** | T***N**,F***N**,Y***I**,P***M**,G***I** | K****H**,*N******N***,D****K**,Q****M**,M****N** | *T****R****E*,S**S**V,K**E**A,Q**R**L,T**D**A | **I**V,**V**K,**I**I,**T**K,**M**V | **I***D,**M***V,**N***Q,**T***N,**H***N | **S****K,**I****D,***I******M*,**P****N,**Q****A |
| **SKCM** | H**I**,C**I**,Q**Y**,V**I**,T**N** | G***I**,S***C**,H***V**,R***I**,D***I** | S****N**,G****I**,P****I**,I****I**,N****I** | A**A**S,T**D**A,T**V**D,K**A**S,P**V**T | **N**K,**N**E,**Y**R,**I**P,**N**V | **K***Q,**I***Q,**N***N,**V***H,**V***N | **I****L,**I****A,***I******M*,**I****V,**I****Q |
| **HNSC** | N**I**,D**N**,L**Y**,N**M**,I**F** | E***Q**,F***K**,I***N**,F***I**,H***H** | K****N**,H****A**,*N******N***,E****M**,S****N** | K**P**S,P**P**A,K**A**S,S**P**A,T**S**Q | **M**V,***N****Q*,**Q**P,**S**N,**I**C | **Q***L,**N***N,**T***N,**H***D,**Y***S | **I****V,**N****H,**N****D,**M****G,**I****K |
| **Others** | G**L**,E**M**,H**D**,N**I**,Q**F** | S***F**,F***I**,R***M**,D***I**,Y***H** | N****M**,F****I**,V****H**,Y****V**,E****M** | *T****R****E*,K**P**S,P**R**A,P**V**S,A**T**S | **M**Q,**C**S,**H**V,**I**N,**F**G | **N***E,**H***Y,**V***G,**L***W,**M***Q | **I****V,***I******M*,**H****F,**H****H,**M****F |

**Table S7b:** Preferred motifs in passengers across cancer types

M: Mutation site; *:gap and X represents any residue present in N- or C-termini.

Amino acid presented in the bold represent the site of mutation. Motifs presented in the italics are observed in at least three cancer types.

**Table S8:** Important features across cancer types

| **Group** | **Total Number of Features** | **Sequence-based** | **Structure-based** | **Network-based** |
| --- | --- | --- | --- | --- |
| Hem.Lymph | 29 | Di, Tri-peptide motifs, Conformational, Energetic, Composition, Conservation, PSSM | ASA, Disorder, Disulphide, Residue depth, AA predicted local distance difference test | Degree, Closeness, Betweenness, Eigenvector |
| BRCA | 35 | Tri-peptide motifs, Conformational, Physical, Mutation matrices, Composition, Conservation | Cation-pi interaction, aromatic interaction, Disorder, Residue depth, predicted local distance difference test | Degree |
| COADREAD | 37 | Conformational, Physical, Conservation | Aromatic, Aromatic-sulphur, predicted local distance difference test, secondary structure | - |
| STAD | 31 | Di, Tri-peptide motifs, Chemical, Energetic, Composition, Mutation matrices, Conservation | Disorder, Disulphide, Residue depth, secondary structure | - |
| Digestive | 26 | Di, Tri-peptide motifs, Physical, Chemical, Conformational, Energetic, Conservation, PSSM | Disorder, secondary structure, ASA, Residue depth | - |
| Kidney | 25 | Di, Tri-peptide motifs, Conformational, Mutation matrices, Chemical, Composition | Predicted local distance difference test, Residue depth, ASA, secondary structure | Degree |
| BLCA | 26 | Di, Tri-peptide motifs, Conformational, Chemical, Composition | ASA, predicted local distance difference test, secondary structure | - |
| LUAD | 25 | Di, Tri-peptide motifs, Conformational, Energetic, Composition, Conservation | Cation-pi, Aromatic-sulphur, AA predicted local distance difference test, Residue depth | Degree, Closeness, Betweenness |
| Thoracic | 26 | Di, Tri-peptide motifs, Conformational, Energetic, Mutation matrices, Conservation, PSSM | Disorder, Disulfide, Residue depth, secondary structure | Degree |
| Gynaecologic | 30 | Di, Tri-peptide motifs, Chemical, Conformational, Composition | Residue depth, AA predicted local distance difference test, ASA, secondary structure | Degree |
| Endocrine | 29 | Di, Tri-peptide motifs, Conformational, Energetic, Chemical, Mutation matrices, Composition, Conservation | Cation-pi, Aromatic-sulphur**,** Disulfid**e**, Residue depth | Closeness, Betweenness, Eigenvector |
| SKCM | 27 | Di, Tri-peptide motifs, Chemical, Conformational, Composition, Conservation, PSSM | ASA, predicted local distance difference test, | Closeness |
| HNSC | 25 | Di, Tri-peptide motifs, Conformational, Energetic, Composition, Conservation | Disorder, Disulphide, Residue depth, ASA | Degree |
| UCEC | 31 | Di, Tri-peptide motifs, Conformational, Mutation matrices, Physical, Energetic, Conservation | AA predicted local distance difference test, polar, Sec structure, Side chain | - |
| Others | 35 | Di, Tri-peptide motifs, Conformational, Chemical, Conservation | ASA, aromatic, aromatic-sulphur, Cation-pi | Degree, Closeness, Betweenness, Eigenvector |

**Table S9:** Performance of models for mutations in proteins, which are not in the training set

| **Cancer group** | **Driver** | **Passenger** | **Proteins** | **Accuracy** | **Sensitivity** | **Specificity** | **AUC** | **MCC** |
| --- | --- | --- | --- | --- | --- | --- | --- | --- |
| **hem_lymph** | 595 | 328 | 65 | 87.00 | 85.55 | 89.63 | 0.876 | 0.731 |
| **BRCA** | 881 | 772 | 99 | 80.88 | 77.75 | 84.46 | 0.811 | 0.621 |
| **COADREAD** | 2921 | 1020 | 123 | 82.56 | 81.44 | 85.78 | 0.836 | 0.614 |
| **STAD** | 767 | 605 | 76 | 82.29 | 85.14 | 78.68 | 0.819 | 0.640 |
| **digestive** | 821 | 843 | 78 | 84.62 | 74.18 | 94.78 | 0.845 | 0.706 |
| **kidney** | 270 | 699 | 64 | 86.69 | 81.48 | 88.70 | 0.851 | 0.681 |
| **BLCA** | 694 | 461 | 84 | 84.24 | 80.69 | 89.59 | 0.851 | 0.689 |
| **LUAD** | 1305 | 286 | 84 | 80.83 | 79.92 | 84.97 | 0.824 | 0.535 |
| **thoracic** | 826 | 567 | 68 | 86.07 | 79.90 | 95.06 | 0.875 | 0.737 |
| **gynecologic** | 1403 | 785 | 92 | 78.88 | 71.56 | 91.97 | 0.818 | 0.610 |
| **UCEC** | 1521 | 883 | 85 | 84.36 | 89.22 | 75.99 | 0.826 | 0.660 |
| **endocrine** | 451 | 533 | 86 | 85.06 | 83.37 | 86.49 | 0.849 | 0.699 |
| **SKCM** | 2352 | 928 | 111 | 87.90 | 92.05 | 77.37 | 0.847 | 0.700 |
| **HNSC** | 430 | 387 | 74 | 81.88 | 84.65 | 78.81 | 0.817 | 0.636 |
| **Others** | 1094 | 772 | 105 | 79.64 | 85.74 | 70.98 | 0.784 | 0.576 |

**Supplementary Figures**


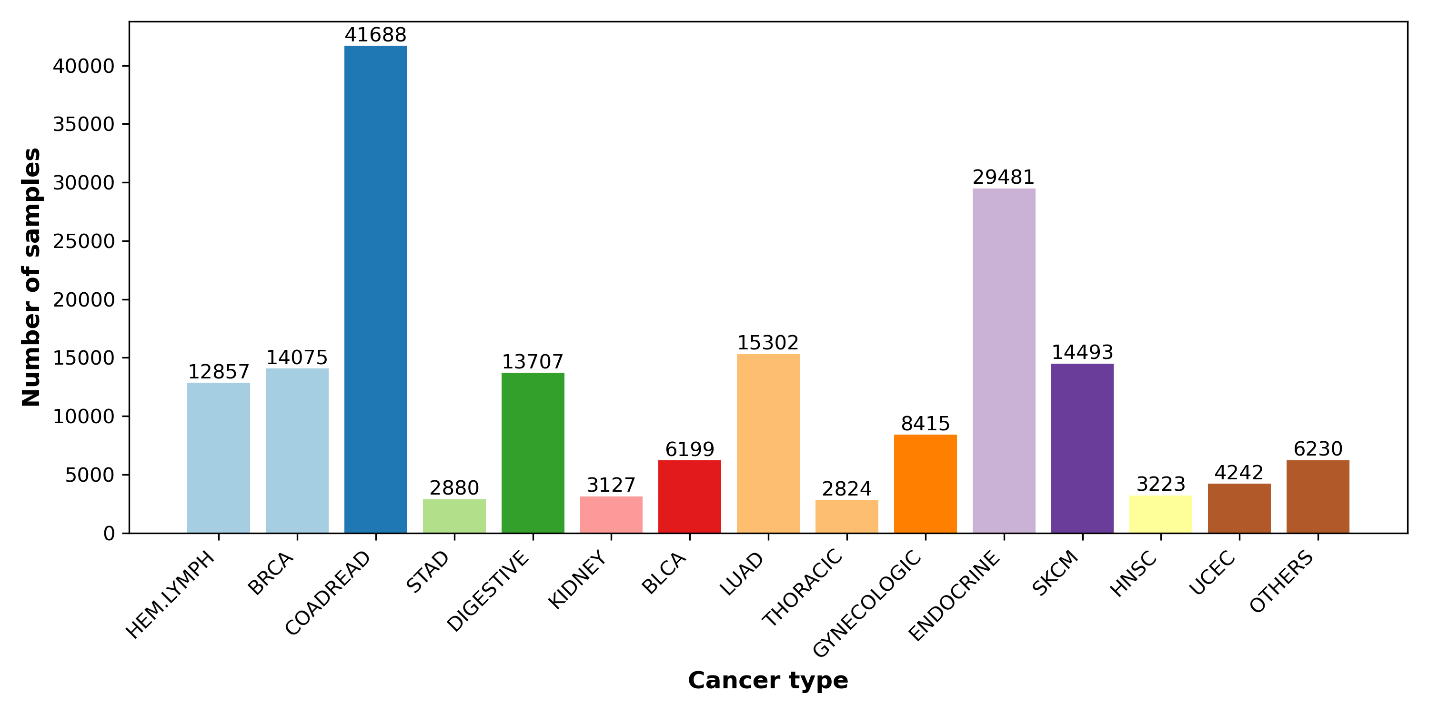


**Figure S1:** Number of samples in COSMIC (v97) for somatic mutations in different cancer types


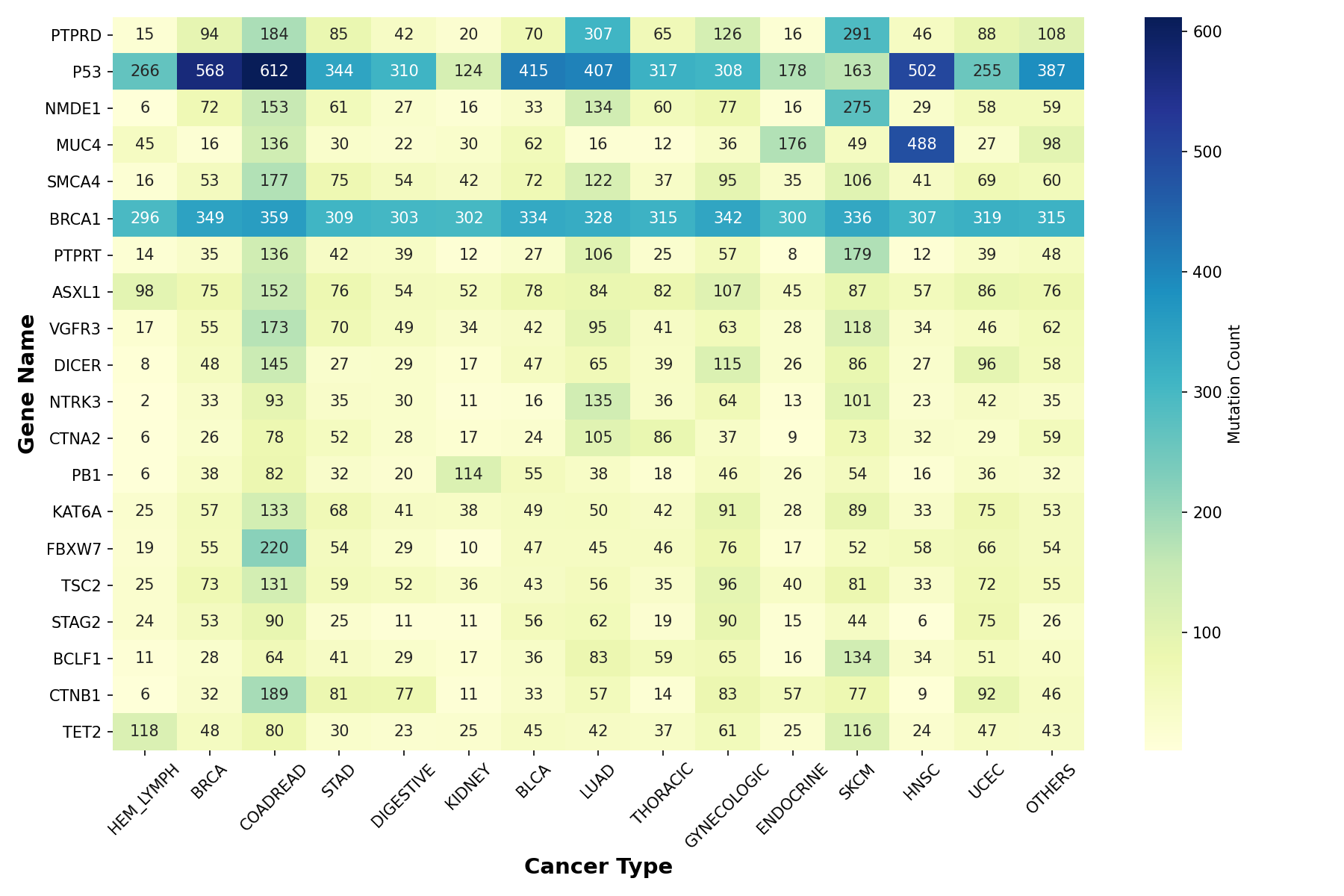


**Figure S2:** Distribution of driver mutations in top 20 proteins preferred across cancer types


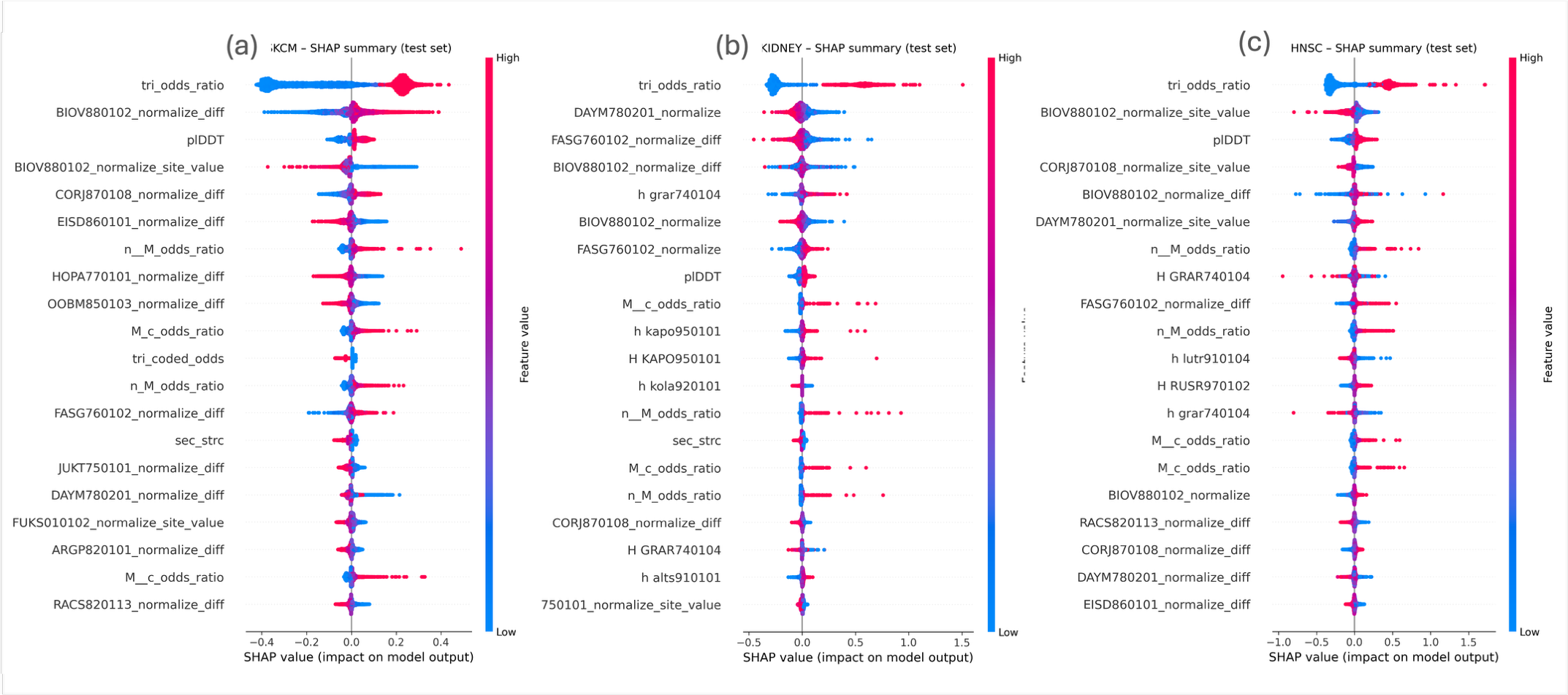


**Figure S3:** SHAP summary plot for top 20 features in different cancer types (a) SKCM, (b) KIDNEY, and (c) HNSC


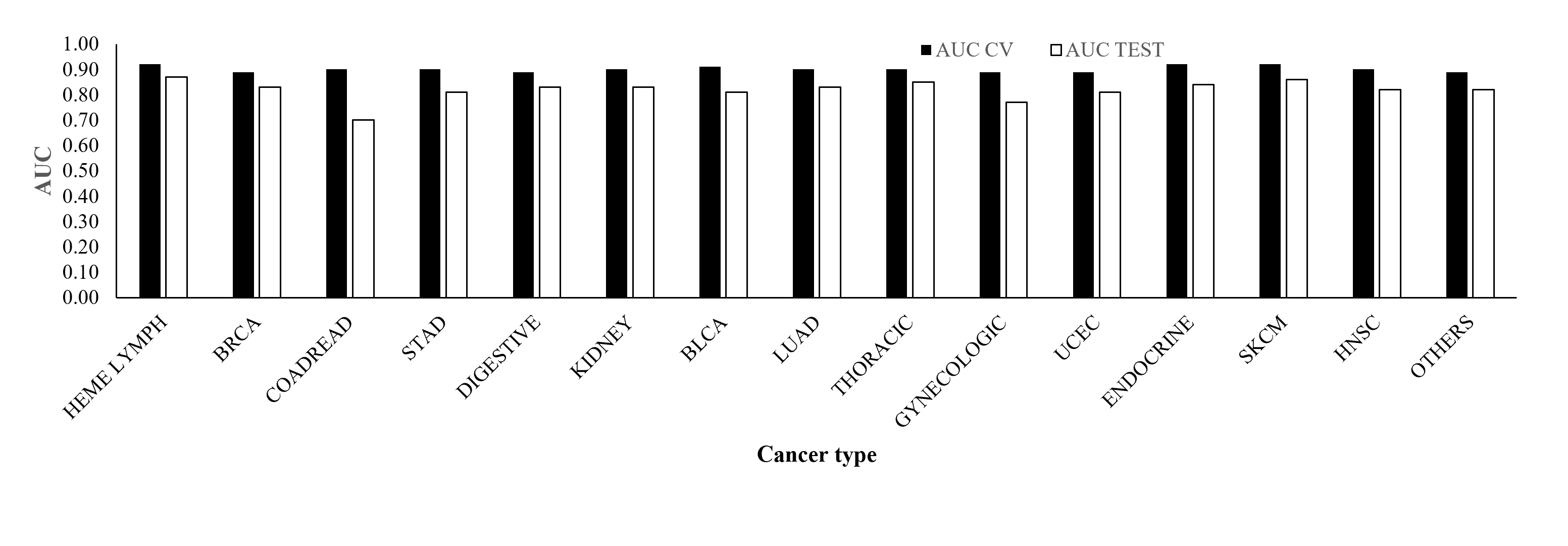


**Figure S4:** Area under the ROC curve (AUC) on 10-fold cross-validation and test set across cancer types


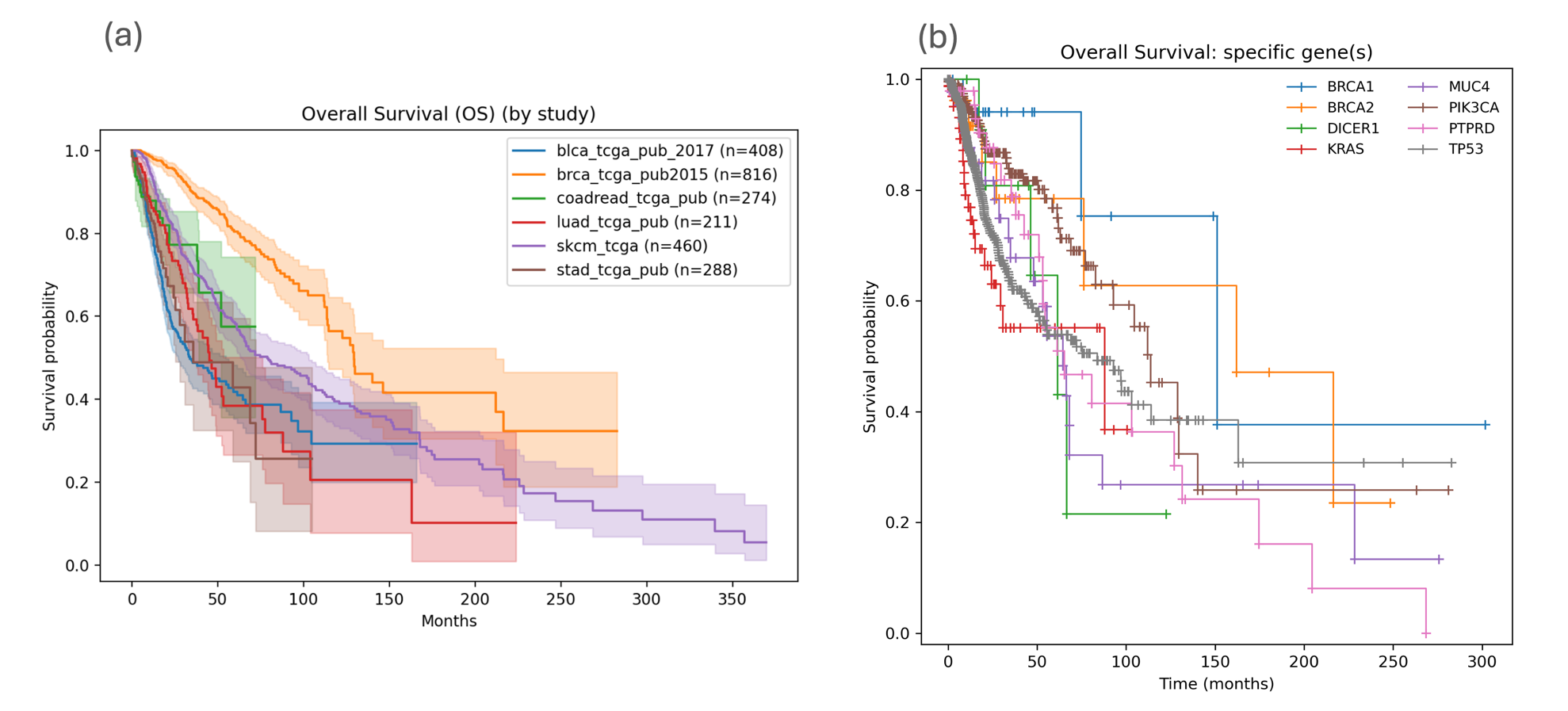


**Figure S5:** Overall survival analysis performed on (a) TCGA clinical datasets for individual cancer types and (b) specific genes with higher driver mutations
